# Analysis of immune and autophagy-related genes and their regulatory mechanisms in osteoporosis patients post-menopause

**DOI:** 10.3389/fendo.2026.1783374

**Published:** 2026-06-01

**Authors:** Zhanfeng Zhang, Jianyou Li, Kuan Ni, Chengyin Lei, Xining Li, Yunliang Yao

**Affiliations:** 1 Department of Orthopedics, The First Affiliated Hospital of Huzhou Normal University, Huzhou, Zhejiang, China; 2 Huzhou Key Laboratory of Early Diagnosis and Treatment of Osteoarthritis, Huzhou, Zhejiang, China; 3 Department of Orthopedics, Huzhou Central Hospital, Affiliated Central Hospital of Huzhou Normal University, Huzhou, Zhejiang, China; 4 Key Laboratory of Vector Biology and Pathogen Control of Zhejiang Province, Huzhou Key Laboratory of Precise Prevention and Control of Major Chronic Diseases, Huzhou Normal University, Huzhou, Zhejiang, China

**Keywords:** autophagy, diagnostic model, immune cell, postmenopausal osteoporosis, WGCNA

## Abstract

**Background:**

Postmenopausal osteoporosis (PMO) is a prevalent metabolic bone disease wherein immune dysregulation and autophagy are critically involved. This study aimed to identify key genes linking immune cells and autophagy in PMO and construct a diagnostic model.

**Methods:**

Autophagy-immune-related differentially expressed genes (DEGs) were identified by integrating limma analysis, WGCNA module genes, and autophagy-related genes (ARGs). Hub genes were screened via protein-protein interaction (PPI) network and statistical tests. A diagnostic model was built and validated. Functional enrichment (GSEA) and immune infiltration analysis (GSVA) were performed. Regulatory networks involving transcription factors (TFs) and microRNAs (miRNAs) were constructed. Drug prediction and single-cell RNA sequencing (scRNA-seq) analyses were conducted. An ovariectomized (OVX) mouse model was established to assess bone mineral density, serum biomarkers, and hub gene expression via RT-qPCR.

**Results:**

Three hub genes (CDK2, DDIT3, MAPK8) were identified. The diagnostic model exhibited high predictive accuracy (AUC = 0.91). GSEA indicated CDK2 and DDIT3 were associated with ligand-receptor interaction pathways. Hub genes correlated significantly with altered immune cell infiltration. Regulatory networks included multiple TFs and miRNAs. Drug prediction identified potential therapeutics targeting each hub gene. scRNA-seq highlighted bone marrow mesenchymal stem cells (BM MSCs) as a key site, with pseudotemporal analysis revealing dynamic hub gene expression during differentiation. *In vivo*, OVX mice showed reduced bone density, estradiol, and osteocalcin, elevated CTX-1, and increased MAPK8 and DDIT3 expression.

**Conclusion:**

An immune-autophagy-related diagnostic model based on DDIT3 and MAPK8 was developed and validated, offering new insights into PMO evaluation and therapeutic strategy.

## 1 Introduction

Postmenopausal Osteoporosis (PMO) is a condition that affects the skeleton, leading to lower bone density and the weakening of bone structure, particularly in women post-menopause (1). The decline in estrogen levels during menopause significantly increases the risk of PMO. Estrogen is essential for maintaining bone density (2). Additional risk factors include older age, genetic factors, low body weight, smoking, and a lack of physical activity (3). PMO’s pathophysiology features an imbalance between bone resorption and formation, primarily caused by heightened osteoclast activity resulting from estrogen deficiency (4–6). This increase of osteoclastogenesis leads to excessive bone loss, resulting in weakened bones that are highly susceptible to fractures (7). Common pathological features include microarchitectural disruption of trabecular bone and cortical thinning (8, 9). Currently, treatment options for PMO include bisphosphonates, hormone replacement therapy (HRT), and selective estrogen receptor modulators (SERMs) (10–12). Although these treatments can lower fracture risk, they have limitations. Bisphosphonates may lead to gastrointestinal issues and long-term safety concerns, while hormone replacement therapy (HRT) carries risks of cardiovascular problems and certain cancers. Therefore, there remains a pressing need for more effective and safer therapeutic strategies to manage PMO and improve patient outcomes.

At the cellular level, the immune system and the autophagy process form a tightly interconnected regulatory network in the pathogenesis of Postmenopausal Osteoporosis (PMO). Autophagy, as a crucial mechanism for the degradation and recycling of damaged cellular components, not only maintains cellular homeostasis but also directly regulates the activation and functional states of immune cells (13). Specifically, the chronic inflammatory environment induced by estrogen deficiency can activate immune cells such as T cells, B cells, and myeloid cells, leading to the release of pro-inflammatory factors like TNF-αand IL-6. These factors, in turn, inhibit osteoblast autophagy and enhance osteoclast autophagy primarily through the mTOR and NF-κB signaling pathways, resulting in decreased bone formation and increased bone resorption (14–17). Conversely, dysfunctional autophagy can promote the pro-inflammatory polarization of immune cells, exacerbating the inflammatory response within the bone microenvironment and forming a vicious cycle of “immune activation-autophagy dysregulation-bone metabolic imbalance” (18, 19). In recent years, with the advancement of bioinformatics, several studies have utilized the Gene Expression Omnibus (GEO) database to screen for potential biomarkers of PMO. For instance, Fang et al. identified immune-related biomarkers in PMO through methods such as differential expression analysis and machine learning (20). Liu et al. constructed a diagnostic model based on oxidative stress-related genes (21). However, most of these studies are confined to a single omics approach or a specific pathway, and there is still a lack of in-depth exploration that systematically integrates genes at the intersection of immunity and autophagy.

Therefore, this study innovatively integrates multiple bioinformatics strategies to identify key genes at the intersection of immunity and autophagy from multiple dimensions, including immune cell infiltration, weighted gene co-expression network analysis (WGCNA), and protein-protein interaction (PPI) networks. Subsequently, a diagnostic model was constructed. Furthermore, single-cell transcriptomic analysis was employed to dissect the cell-specific expression patterns of these genes within the bone marrow microenvironment. This comprehensive approach aims to provide novel insights into elucidating the molecular mechanisms of PMO and developing targeted therapeutic strategies.

## 2 Materials and methods

### 2.1 Data source and experimental animals

The osteoporosis (OP) related transcriptomic and single-cell RNA sequencing (scRNA-seq) datasets, GSE56815 (22), GSE7158 (23), and GSE147287 datasets, were collected from Gene Expression Omnibus (GEO). Both datasets were derived from human studies. Specifically, GSE56815 (GPL96 platform) included 20 premenopausal low BMD samples (OP group) and 20 premenopausal high BMD samples (normal control (NC) group), while GSE7158 (GPL570 platform) comprised 12 premenopausal low BMD samples (OP group) and 14 premenopausal high BMD samples (NC group). Notably, the sample type in both GSE56815 and GSE7158 databases was human peripheral blood monocytes. For scRNA-seq analysis, the GSE147287 dataset (GPL24676 platform) was used, consisting of 1 OP bone marrow monocyte sample, this sample was from a 67-year-old postmenopausal female patient with OP. The autophagy-related genes (ARGs) were sourced from public databases. Specifically, we obtained 743 ARGs from the Autophagy Gene List (http://www.tanpaku.org/autophagy/list/GeneList.html) (24) and 231 ARGs from the Human Autophagy Database (https://autophagy.lu/v1/clustering/index.html) (25). After merging and removing duplicates, a total of 847 unique ARGs were compiled for subsequent analysis (**Table 1**).

**Table 1:** List of merged and deduplicated autophagy-related genes (ARGs).

AMBRA1	CCR2	GRID1	NRG1	TNFSF10	UGT2B17	TGFBRAP1	SLC3A1	RPS6KA4	PPP2R4	PAK3	MTMR4	KRT74	HCK	EI24	CAPN5	AP2B1
APOL1	CD46	GRID2	NRG2	TP53	UGT2B15	TFEB	SLC36A4	RPS6KA3	PPP2R3C	PAK2	MTMR2	KRT73	HARBI1	EEF1A2	CAPN3	AP2A2
ARNT	CDKN1A	HDAC1	NRG3	TP53INP2	UGT2B10	TFE3	SLC36A1	RPS6KA1	PPP2R3B	PAK1	MTMR1	KRT72	GRB2	EEF1A1	CAPN14	AP2A1
ARSA	CDKN1B	HDAC6	P4HB	TP63	UGT2A3	TEP1	SLC1A7	RIPK4	PPP2R3A	PA2G4	MTM1	KRT71	GRAMD1B	E2F2	CAPN13	AP1S3
ARSB	CDKN2A	HGS	PARK2	TP73	UGT2A1	TELO2	SLC1A6	RIPK1	PPP2R2D	OPHN1	MITF	KRT6C	GLIPR2	E2F1	CAPN12	AP1S2
ATF4	CFLAR	HIF1A	PARP1	TSC1	UGT1A9	TECPR1	SLC1A5	RICTOR	PPP2R2C	OPA1	MINK1	KRT6B	GFAP	DRAM2	CAPN11	AP1S1
ATF6	CHMP2B	HSP90AB1	PEA15	TSC2	UGT1A6	TBC1D7	SLC1A4	RGL1	PPP2R2B	NVL	MFN2	KRT6A	GBF1	DNPEP	CAMKK1	AP1M2
ATG10	CHMP4B	HSPA5	PELP1	TUSC1	UGT1A10	TBC1D5	SLC1A2	RET	PPP2R2A	NUAK2	MFN1	KRT5	GAPDHS	DNM3	CAMK2G	AP1M1
ATG12	CLN3	HSPA8	PEX14	ULK1	UGT1A1	TBC1D4	SLC1A1	RELB	PPP2R1B	NUAK1	MERTK	KRAS	GABARAPL3	DNM2	CAMK2D	AP1G2
ATG16L1	CTSB	HSPB8	PEX3	ULK2	UBE2N	TBC1D25	SIPA1L3	REL	PPP2R1A	NTRK2	MELK	KPNA6	FUNDC1	DNM1L	CAMK2B	AP1G1
ATG16L2	CTSD	IFNG	PIK3C3	ULK3	UBE2L3	TBC1D14	SIN3B	RCAN1	PPP2CB	NRBF2	MARK4	KLHL8	FKBP8	DNM1	CAMK1G	AP1B1
ATG2A	CTSL1	IKBKB	PIK3R4	USP10	UBE2J2	TBC1D12	SIN3A	RALGAPA2	PPP2CA	NRAS	MARK3	KLHL5	FIS1	DES	CAMK1D	AP1AR
ATG2B	CX3CL1	IKBKE	PINK1	UVRAG	UBE2H	SUPT20H	SIM2	RALGAPA1	PPP1CC	NPAS3	MARK2	KLHL4	FGR	DEPTOR	CAMK1	ANKRD44
ATG3	CXCR4	IL24	PPP1R15A	VAMP3	UBE2D3	STXBP3	SIK3	RALB	PPP1CA	NPAS2	MARK1	KLHL28	FGFR3	DDIT4	BRSK2	ANKRD28
ATG4A	DAPK1	IRGM	PRKAB1	VAMP7	UBE2D2	STXBP2	SIK2	RALA	POC1B	NOD2	MAPKAP1	KLHL20	FGFR2	DAW1	BRSK1	ANKK1
ATG4B	DAPK2	ITGA3	PRKAR1A	VEGFA	UBE2A	STXBP1	SIK1	RABGEF1	POC1A	NOD1	MAPK7	KLHL2	FGFR1	DAPK3	BRAF	ANK3
ATG4C	DDIT3	ITGA6	PRKCD	WDFY3	UBC	STX8	SIDT2	RAB9A	PNCK	NLRP9	MAPK15	KLHL18	FBXW7	CTSV	BLK	ANK2
ATG4D	DIRAS3	ITGB1	PRKCQ	WDR45	UBB	STX7	SIDT1	RAB8A	PLD2	NLRP8	MAPK14	KLHL17	EXOC8	CTSL	BECN2	ANK1
ATG5	DLC1	ITGB4	PTEN	WDR45L	TRRAP	STX17	SHC4	RAB5C	PLD1	NLRP7	MAPK10	KLHL13	EXOC7	CTSE	BDNF	ALPPL2
ATG7	DNAJB1	ITPR1	PTK6	WIPI1	TRAPPC9	STK4	SHC3	RAB5B	PLCH2	NLRP5	MAP4K5	KLHL12	EXOC6B	CSNK2B	BCR	ALPP
ATG9A	DNAJB9	KIAA0226	RAB11A	WIPI2	TRAPPC8	STK38L	SHC2	RAB1B	PLCH1	NLRP4	MAP4K3	KLHL1	EXOC6	CSNK2A2	AXL	ALPL
ATG9B	DRAM1	KIAA0652	RAB1A	ZFYVE1	TRAPPC6B	STK36	SHC1	RAB12	PLCG1	NLRP3	MAP4K2	KIAA0226L	EXOC5	CSNK2A1	AURKA	ALPI
ATIC	EDEM1	KIAA0831	RAB24	YME1L1	TRAPPC6A	STK26	SH3GLB2	RAB10	PLCD1	NLRP14	MAP4K1	KEAP1	EXOC4	CSNK1G2	ATR	ALK
BAG1	EEF2	KIF5B	RAB33B	YES1	TRAPPC5	STK25	SH3GL3	PRR5L	PLCB4	NLRP13	MAP3K6	KAT8	EXOC3L4	CSNK1D	ATM	AKT3
BAG3	EEF2K	KLHL24	RAB5A	WDR5B	TRAPPC4	STK24	SH3GL2	PRR5	PKN3	NLRP12	MAP3K14	KAT7	EXOC3L1	CSNK1A1L	ATG14	AKT2
BAK1	EGFR	LAMP1	RAB7A	WDR5	TRAPPC3	SRC	SH3GL1	PRMT8	PKN2	NLRP1	MAP2K5	KAT6B	EXOC3	CLTC	ATG13	AKT1S1
BAX	EIF2AK2	LAMP2	RAC1	WDR45B	TRAPPC2	SPG7	SGK2	PRMT3	PKN1	NLRC5	MAP2K4	KAT6A	EXOC2	CLTB	ATG101	AKT1
BCL2	EIF2AK3	MAP1LC3A	RAF1	WDR3	TRAPPC10	SPATA5L1	SESN3	PRMT1	PIP5K1C	NLRC3	MAP2K3	KAT5	EXOC1	CLTA	ARNTL2	AGFG1
BCL2L1	EIF2S1	MAP1LC3B	RB1	WDFY4	TRAPPC1	SPATA5	SESN1	PRKDC	PIP5K1B	NFKBIZ	MAP2K2	JUN	EVA1A	CLOCK	ARNTL	AFG3L2
BECN1	EIF4EBP1	MAP1LC3C	RB1CC1	WAC	TRAF6	SPATA13	SEC24D	PRKCZ	PIP5K1A	NFKBIE	MAP2K1	ITPR3	ERN2	CLEC16A	ARNT2	AES
BID	EIF4G1	MAP2K7	RELA	VTI1B	TRAF5	SOS2	SEC24C	PRKCI	PIP4K2A	NFKBID	MAN2C1	ITPR2	ERBB4	CHUK	ARL2	ACTR3B
BIRC5	ERBB2	MAPK1	RGS19	VPS45	TRAF4	SOS1	SEC24B	PRKCH	PIK3CG	NFKBIB	MAN2B1	IRS4	ERBB3	CELF5	ARHGEF9	ACTR3
BIRC6	ERN1	MAPK3	RHEB	VPS41	TRAF3	SOD2	SEC23B	PRKCG	PIK3CD	NFKBIA	MAN2A2	IRS2	EPN3	CELF3	ARHGEF4	ACTR2
BNIP1	ERO1L	MAPK8	RPS6KB1	VPS39	TRAF2	SOD1	SEC23A	PRKCE	PIK3CB	NFKB2	LYST	IRS1	EPN2	CELF1	ARHGEF17	ACTG2
BNIP3	FADD	MAPK8IP1	RPTOR	VPS33B	TRAF1	SNX4	SEC16B	PRKCB	PIK3CA	NFE2L3	LRSAM1	INSRR	EPN1	CDKL5	ARHGAP42	ACTG1
BNIP3L	FAM48A	MAPK9	SAR1A	VPS33A	TRADD	SNX30	SEC16A	PRKCA	PIK3C2B	NFE2L1	LRBA	INSR	EPHB3	CDKL4	ARHGAP26	ACTC1
C12orf44	FAS	MBTPS2	SERPINA1	VPS18	TPTE2	SNX1	SCOC	PRKACB	PIK3C2A	NCOA4	LPIN2	INS	EPHB2	CDKL1	ARHGAP10	ACTB
C17orf88	FKBP1A	MLST8	SESN2	VPS16	TPR	SNRNP40	SBF2	PRKACA	PI4KB	NBEA	LPIN1	IGF1R	EPHB1	CDK5	ARFGEF2	ACTA2
CALCOCO2	FKBP1B	MTMR14	SH3GLB1	VPS13C	TOR1B	SNRK	SAR1B	PRKAB2	PGAM5	NAPSA	LCK	IARS	EPHA5	CDK2	ARFGEF1	ACTA1
CAMKK2	FOS	MTOR	SIRT1	VPS13A	TOMM7	SNAP29	RYR2	PRKAA2	PGA5	MYO3A	LATS2	HUNK	EPHA4	CDK18	ARF6	ACBD5
CANX	FOXO1	MYC	SIRT2	VMP1	TNS3	SMPDL3B	RYR1	PRKAA1	PGA4	MYLK3	KSR1	HSPA6	EPHA3	CDK17	ARAF	ACAP3
CAPN1	FOXO3	NAF1	SPHK1	VIM	TNS2	SMPD1	RUNX3	PREX2	PGA3	MYLK	KRT85	HSPA2	EPHA2	CDK16	APAF1	ACAP2
CAPN10	GAA	NAMPT	SPNS1	VDAC3	TNS1	SLC7A9	RUNX1	PREX1	PEX5L	MYD88	KRT84	HSPA1L	EPG5	CDK13	AP4M1	ABR
CAPN2	GABARAP	NBR1	SQSTM1	VDAC2	TNIK	SLC7A8	RUBCN	PPP6C	PEX5	MX2	KRT83	HSPA1B	EPAS1	CDK11A	AP4E1	ABL2
CAPNS1	GABARAPL1	NCKAP1	ST13	VDAC1	TNFAIP2	SLC7A7	RRAGD	PPP4C	PEX1	MX1	KRT82	HSPA1A	EIF4EBP3	CDK1	AP4B1	ABL1
CASP1	GABARAPL2	NFE2L2	STK11	VCP	TMEM173	SLC7A6	RRAGC	PPP2R5E	PDPK1	MTRF1L	KRT81	HRAS	EIF4EBP2	CAPNS2	AP3M2	ABCB6
CASP3	GAPDH	NFKB1	TBK1	VAMP8	TM9SF4	SLC7A5	RRAGB	PPP2R5D	PDK1	MTMR9	KRT8	HIF3A	EIF4E1B	CAPN9	AP3M1	Mb3875
CASP4	GNAI3	NKX2-3	TM9SF1	UGT2B7	TM9SF3	SLC7A11	RRAGA	PPP2R5C	PAK7	MTMR8	KRT79	HDAC7	EIF4E	CAPN8	AP3D1	
CASP8	GNB2L1	NLRC4	TMEM49	UGT2B4	TM9SF2	SLC7A10	RPS6KB2	PPP2R5B	PAK6	MTMR7	KRT76	HDAC5	EIF2AK4	CAPN7	AP2S1	
CCL2	GOPC	NPC1	TMEM74	UGT2B28	TIGAR	SLC3A2	RPS6KA6	PPP2R5A	PAK4	MTMR6	KRT75	HDAC4	EIF2AK1	CAPN6	AP2M1	

Female C57BL/6 mice (6–8 weeks old) were obtained from Hangzhou Ziyuan Experimental Animal Technology Co., Ltd(China) and housed in a controlled environment with a 12-hour light/dark cycle. All animal experiments were approved by the Huzhou University Animal Care and Use Committee.

### 2.2 Differential expression analysis of GSE56815 dataset

Firstly, the differentially expressed genes (DEGs) were extracted via limma package (v3.50.1) in GSE56815 dataset (adj.p-value < 0.05) (26). The enrichment of 28 immune cells in OP and NC specimens was evaluated via GSVA package (v1.42.0) (27). Then, Wilcoxon test was performed to acquire the differential immune cells between OP and NC specimens.

### 2.3 Weighted gene co-expression network analysis

The immune cell-related genes were determined by WGCNA package (v1.70-3) (28). Firstly, to ensure the accuracy of the subsequent analysis, the specimens were clustered to remove the outliers. We used differential immune cells as trait data, the specimens were re-clustered. Then, the optimal soft threshold (β) was determined to ensure that the network approached scale-free distribution. Next, genes were divided into several modules. Finally, the pivotal modules and genes most associated with differential immune cells were determined.

### 2.4 Identification of autophagy-immune cell-related DEGs

We screened immune cell-related DEGs by overlapping DEGs and pivotal module genes acquired from WGCNA. Next, intersecting genes obtained by overlapping immune cell-related DEGs and ARGs were defined as candidate genes. Subsequently, the chromosome localization of candidate genes was achieved via OmicCircos package (v1.32.0) (29).

### 2.5 Functional annotation and ingenuity pathway analysis

The Gene Ontology (GO) and Kyoto Encyclopedia of Genes and Genomes (KEGG) enrichment analysis were implemented via clusterProfiler package (v4.2.2) (adj.p-value < 0.05) (30). Then, the pathway and biological function of candidate genes were investigated by QIAGEN IPA (www.qiagen.com/ingenuity).

### 2.6 Identification of hub genes

The PPI network of candidate genes was constructed via STRING database. Then, critical genes were determined by Cytoscape software (v3.8.2) (31). The expression discrepancy of critical genes between OP and NC specimens in GSE56815 and GSE7158 was researched by Wilcoxon test. In addition, genes with consistent expression trends in two datasets were defined as hub genes. The correlation analysis among hub genes was executed through corrplot package (v0.92) (32). Subsequently, the diagnostic model was built by glm function. Then, based on the screened hub genes, the PRROC package (v1.3.1) (33) was used to plot the receiver operating characteristic (ROC) curves. Similarly, based on the hub genes, a nomogram was constructed using the RMS package (34). Finally, evaluation of the predictive effect of the nomogram was done (DCA curve and calibration curve).

### 2.7 Gene set enrichment analysis

Firstly, the specimens were stratified into low- and high-expression groups in GSE56815 dataset depending on hub genes expression. Then, logFC was calculated via limma package (v3.50.1) (35). The GSEA analysis with reference to MSigDB database was executed according to the logFC order.

### 2.8 The regulatory network of hub genes

The transcription factors (TFs) and miRNAs of hub genes were excavated from miRNet database (https://www.mirnet.ca/miRNet/home.xhtml). Then, Cytoscape software was used for visualization of the network between hub genes and TFs/miRNAs.

### 2.9 Relevance analysis of hub genes and immune cells and drug prediction

The relevance of hub genes and differential immune cells was acquired through psych package (v2.1.6) (https://CRAN.R-project.org/package=psychVersion=2.2.3) (p < 0.05, |cor| > 0.3). Next, the small molecule drugs of hub genes were excavated by DGIdb database.

### 2.10 The scRNA-seq analysis

The scRNA-seq dataset GSE147287 was analyzed using the “Seurat” package (v 5.30) (26). Quality control (QC) was performed to filter out low-quality cells and genes. Specifically, genes expressed in fewer than three cells were excluded. The retained cells met the following criteria: 300 < nFeature_RNA (genes per cell) < 4,000, nCount_RNA (total RNA count per cell) < 20,000, and percent.mt (proportion of mitochondrial gene expression) < 20%. After QC, the data were integrated using the Harmony function and normalized with the NormalizeData function. Highly variable genes (HVGs) were identified using the FindVariableFeatures function, and the data were then scaled with the ScaleData function. Principal component analysis (PCA) was performed on the HVGs using the RunPCA function, and the number of principal components (PCs) to retain was determined based on the results from the JackStrawPlot and ElbowPlot functions (P < 0.05). Unsupervised clustering was performed using the FindNeighbors and FindClusters functions, followed by cluster visualization with t-distributed Stochastic Neighbor Embedding (tSNE) (resolution = 0.2). The specifically differentially expressed genes in each cluster were confirmed by FindAllMarkers function (logfc.threshold=0.25, min.pct=0.25, only.pos=TURE). Cell types were annotated based on the expression of known marker genes from the literature (36) and the CellMarker database (http://www.bio-bigdata.center/CellMarkerSearch.jsp). The proportion of annotated cells in OP sample was analyzed. Additionally, the expression levels of hub genes within each annotated cell were explored. Cells exhibiting high expression of hub genes were identified as key cells.

### 2.11 Pseudo-temporal and cell-to-cell communication analyses

In the GSE147287 dataset, to gain a deeper understanding of the key cells and other annotated cell clusters, cell-to-cell communication analysis was performed using the “CellChat” package (v 1.6.1) (37). Besides, to explore the key cells in more detail, secondary dimensionality reduction was conducted. This involved reapplying the dimensionality reduction method from section 2.10. Next, pseudo-temporal analysis was performed to examine the differentiation states and trajectories of the key cells using the “Monocle” package (v 2.26.0) (38). This analysis revealed dynamic expression patterns of hub genes across the pseudo-temporal progression, providing valuable insights into the developmental trajectories and differentiation states of these cells.

### 2.12 Construction model

The ovariectomy (OVX) was performed to establish a postmenopausal osteoporosis (OP) model. Mice were anesthetized by inhalation of 2% isoflurane (Sigma-Aldrich, USA) in oxygen at a flow rate of 1 L/min. A midline abdominal incision was made, both ovaries were excised, and the incision was closed with sutures. Sham-operated mice underwent the same surgical procedure without ovarian removal. Mice were allowed to recover for 2 weeks before further experiments. All mice used in this study were of the same strain (C57BL/6), sex (female), and consistent age (6–8 weeks) at the time of surgery. They were housed under identical conditions (12-hour light/dark cycle, standard chow, and water ad libitum) and acclimatized for one week prior to any experimental procedures. Mice were randomly allocated from the same cohort into either the sham-operated group or the ovariectomy (OVX) group to ensure baseline comparability.

### 2.13 Histological evaluation

At 3 months post-surgery, mice were euthanized by cervical dislocation under deep anesthesia, and femurs were collected for histological analysis. The bone samples were fixed in 10% formalin (Sigma-Aldrich, USA) for 24 hours and then decalcified in a 10% EDTA solution (Sigma-Aldrich, USA) for 4 weeks. Following decalcification, femurs were embedded in paraffin (Sigma-Aldrich, Germany), sectioned at 5 µm thickness, and stained using Hematoxylin and Eosin (HE) staining (Beyotime Biotechnology, China). Stained sections were examined under a light microscope (Olympus BX53, Olympus Corporation, Japan) to assess changes in bone microarchitecture.

### 2.14 Bone density measurement

Bone mineral density (BMD) of the left femur was measured at 1, 2, and 3 months after ovariectomy (OVX) using a dual-energy X-ray absorptiometry (DXA) system (Discovery WA, Hologic, USA) equipped with small−animal software. Mice were anesthetized and placed in the prone position on the scanning platform, with the hind limbs fixed and aligned symmetrically to ensure consistent scanning position. BMD values were calculated automatically by the system’s built−in analysis software following the manufacturer’s standard protocols, and expressed in mg/cm². All measurements were performed by the same experienced operator in a quiet, temperature−controlled environment to minimize variability. BMD was determined in both the OVX group and the sham−operated group to evaluate the osteopenic effect of OVX.

### 2.15 Serum biochemical analysis

Blood samples were collected from the orbital sinus of the mice at 1, 2, and 3 months post-OVX to evaluate serum markers associated with bone metabolism. Serum was separated by centrifugation at 3000 rpm for 10 minutes at 4 °C and stored at -80 °C until analysis. Estradiol, osteocalcin, and CTX-1 levels were measured using ELISA kits (Shanghai Enzyme-linked Biotechnology Co., Ltd., China). Results were compared between OVX mice and sham-operated controls to confirm the success of the OP model.

### 2.16 RNA extraction and real‐time PCR analysis

At 2 and 3 months post-operation, mice were euthanized by cervical dislocation under deep anesthesia, and peripheral blood was collected via cardiac puncture into EDTA-coated tubes to prevent coagulation. Peripheral blood mononuclear cells (PBMCs) were isolated immediately using a Mouse Peripheral Blood Lymphocyte Separation Kit (Beyotime, Shanghai, China) according to the manufacturer’s protocol. Briefly, the collected whole blood was diluted 1:1 with phosphate-buffered saline (PBS). The diluted blood was carefully layered onto an equal volume of the separation solution and centrifuged at 400×g for 30 minutes at room temperature with the brake turned off. The opaque PBMC layer at the interface was carefully aspirated and transferred to a new tube. The harvested PBMCs were washed twice with PBS through centrifugation at 300×g for 10 minutes to remove platelets and separation solution residue. The final PBMC pellet was used for subsequent RNA extraction. Total RNA was then extracted from the purified PBMCs using Trizol (Beyotime, Shanghai, China). The concentration and purity of the RNA were measured spectrophotometrically. Subsequently, complementary DNA (cDNA) was synthesized from a fixed amount of total RNA using BeyoRT™ II cDNA Synthesis Kit (Beyotime, Shanghai, China). The messenger RNA levels of target genes were assessed by quantitative real-time PCR using BeyoFast™ SYBR Green qPCR Mix (Beyotime, Shanghai, China). Each group included n=6 biological replicates, and each sample was analyzed in triplicate (technical replicates). β-actin was used as the internal control. The primers used in the study were as follows: β-actin, 5′‐AGA GGG AAA TCG TGC GTG AC‐3′ (sense) and 5′‐CAA TAG TGA TGA CCT GGC CGT‐3′ (antisense); CDK2, 5′‐GAG GAG GGA AAT GAG AAC TGG G‐3′ (sense) and 5′‐TTC AGT GAT GTG GCA GCG G‐3′ (antisense); DDIT3, 5′‐CCA GCC CAC TGT TTG TCT CT‐3′ (sense) and 5′‐TTG GTC TTC CAG TGT GTG GG‐3′ (antisense); MAPK8, 5′‐TCT GGC TCA GAA GTT GTT TGA GT‐3′ (sense) and 5′‐GTT TTG ACT GCA AAG TGG TCC T‐3′ (antisense).

### 2.17 Western blot analysis

Total protein was extracted from purified mouse peripheral blood mononuclear cells (PBMCs) using a radioimmunoprecipitation assay (RIPA) lysis buffer (Beyotime, Shanghai, China) supplemented with protease and phosphatase inhibitors. Protein concentration was determined using a BCA protein assay kit (Beyotime, Shanghai, China). Equal amounts of protein (30 μg per lane) were separated by 10% sodium dodecyl sulfate-polyacrylamide gel electrophoresis (SDS-PAGE) and transferred onto polyvinylidene difluoride (PVDF) membranes. After blocking with 5% non-fat dry milk in Tris-buffered saline containing 0.1% Tween-20 (TBST) for 1 h at room temperature, the membranes were incubated overnight at 4 °C with primary antibodies against β-actin, CDK2, DDIT3, and MAPK8, respectively (Beyotime, Shanghai, China). Following three washes with TBST, the membranes were incubated with horseradish peroxidase (HRP)-conjugated secondary antibodies for 1 h at room temperature. Protein bands were visualized using an enhanced chemiluminescence (ECL) detection system (Beyotime, Shanghai, China) and quantified using ImageJ software. β-actin was used as the internal reference for protein loading normalization. Relative protein expression levels were calculated as the ratio of target protein gray value to β-actin gray value. Each group included n=6 biological replicates, and each sample was analyzed in triplicate.

### 2.18 Statistical analysis

All open databases and R software (v4.1.0) were utilized to analyze and visualize in this study. The heat map was painted using pheatmap package (v1.0.12) (39). P < 0.05 was taken as a remarkable discrepancy. In the experimental analysis, GraphPad Prism (v 9.3.1) was employed for statistical analysis and data visualization.

## 3 Results

**Figure 1:**
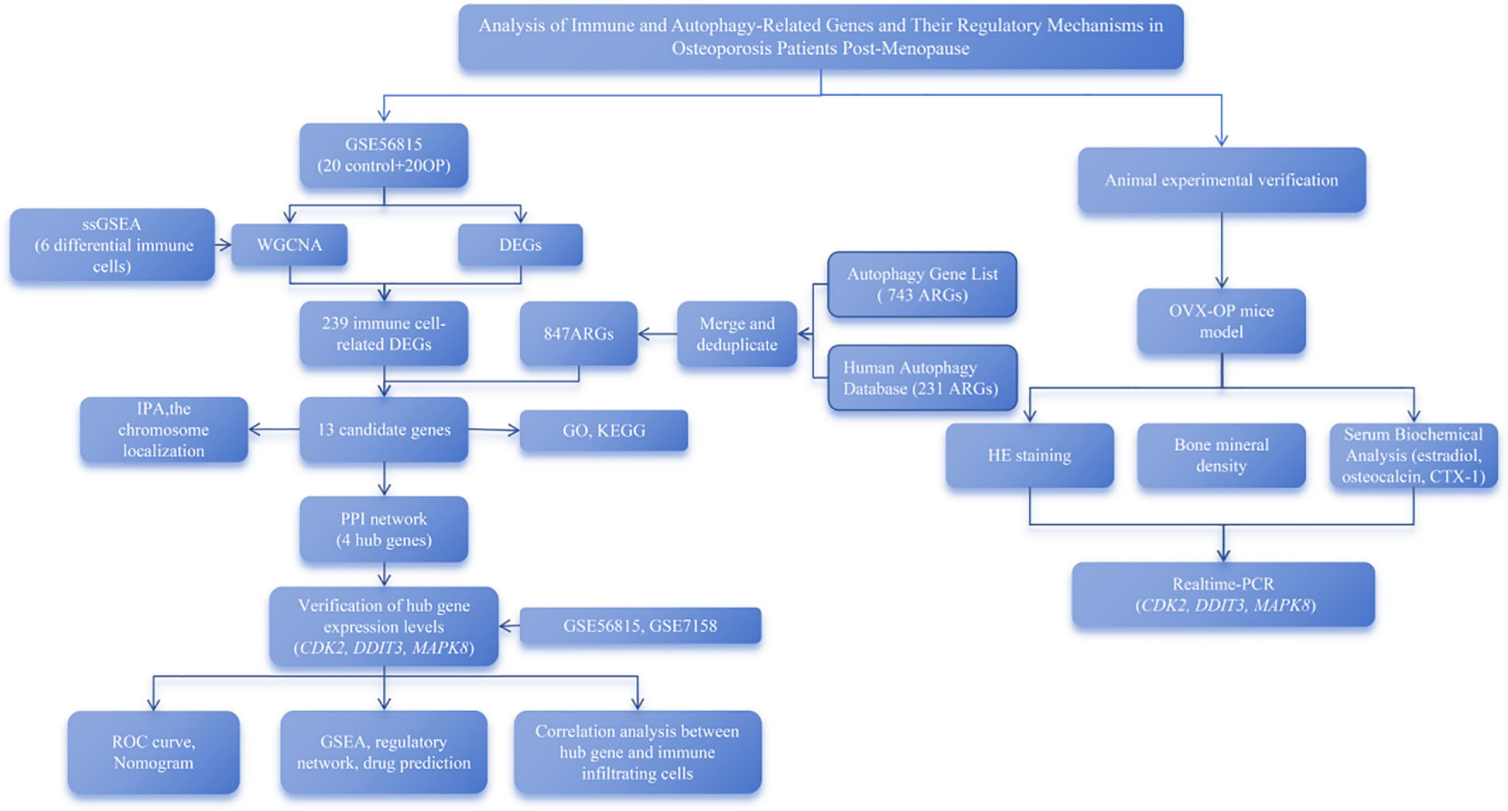
Study workflow diagram.

The specific workflow of this study is shown in **Figure 1**.

### 3.1 The DEGs and differential immune cells in GSE56815 dataset

**Figure 2:**
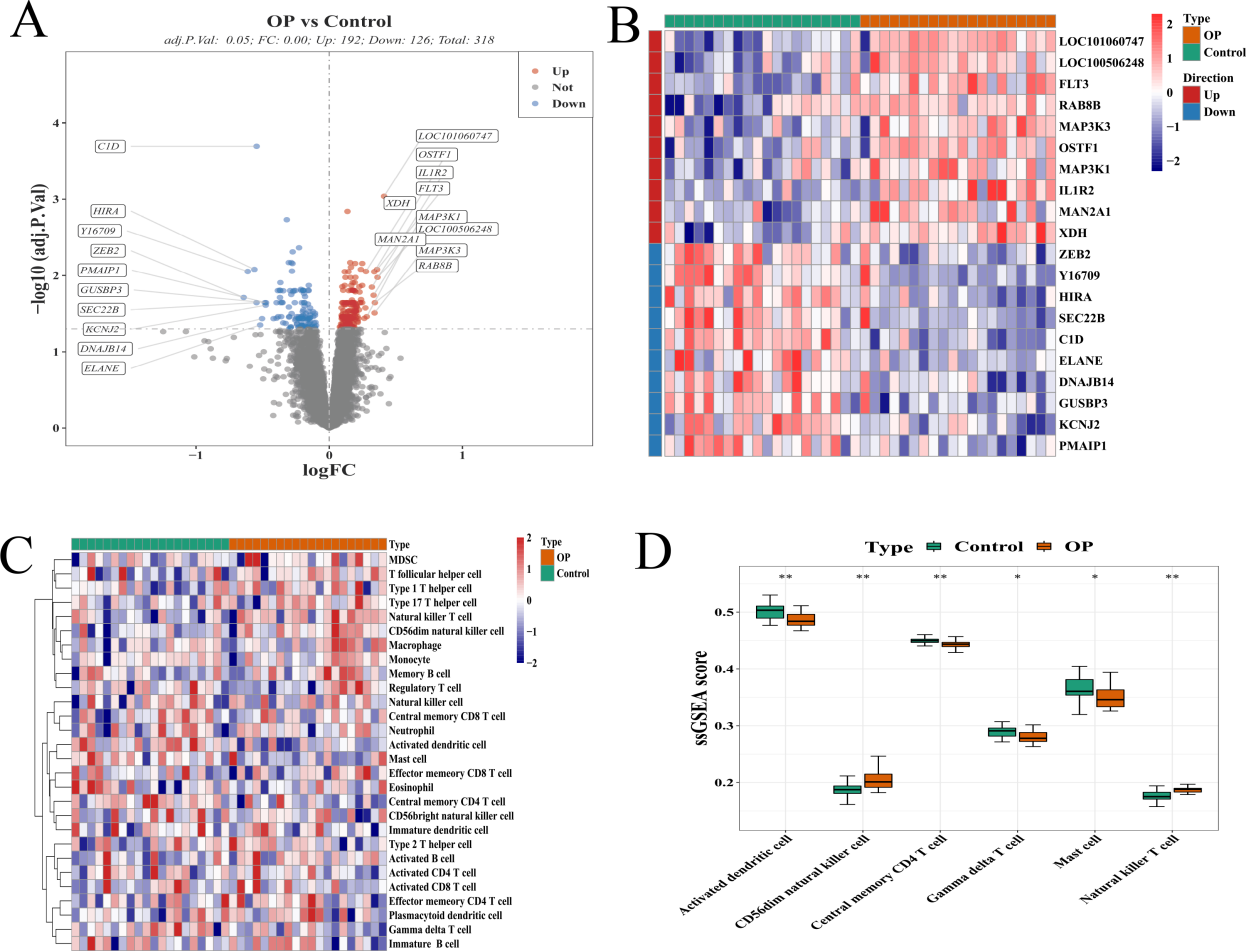
The DEGs and differential immune cells in GSE56815 dataset. **(A)** Gene Distribution Volcano Plot between OP and Control Groups. This analysis was based on gene expression data from human peripheral blood monocytes (Dataset: GSE56815). **(B)** Differential Expression Gene Heatmap between OP and Control Groups. **(C)** Immune Infiltration Cell Enrichment Scores Heatmap between OP and Control Samples. **(D)** The differential immunoinfiltrate cell enrichment fraction was based on the box plot between OP and Control.

A sum of 318 DEGs were acquired in GSE56815 dataset, including 126 down-regulated genes and 192 up-regulated genes in OP specimens (**Figure 2A**). The top10 down- and up-regulated genes were displayed in **Figure 2B**. Moreover, the immune cells in OP and NC specimens were exhibited in **Figure 2C**. Then, 6 differential immune cells in OP specimens (activated dendritic cell, CD56dim natural killer cell, central memory CD4 T cell, gamma delta T cell, mast cell, and natural killer T cell) were determined by Wilcoxon test (**Figure 2D**).

### 3.2 Identification of immune cell-related genes by WGCNA

**Figure 3:**
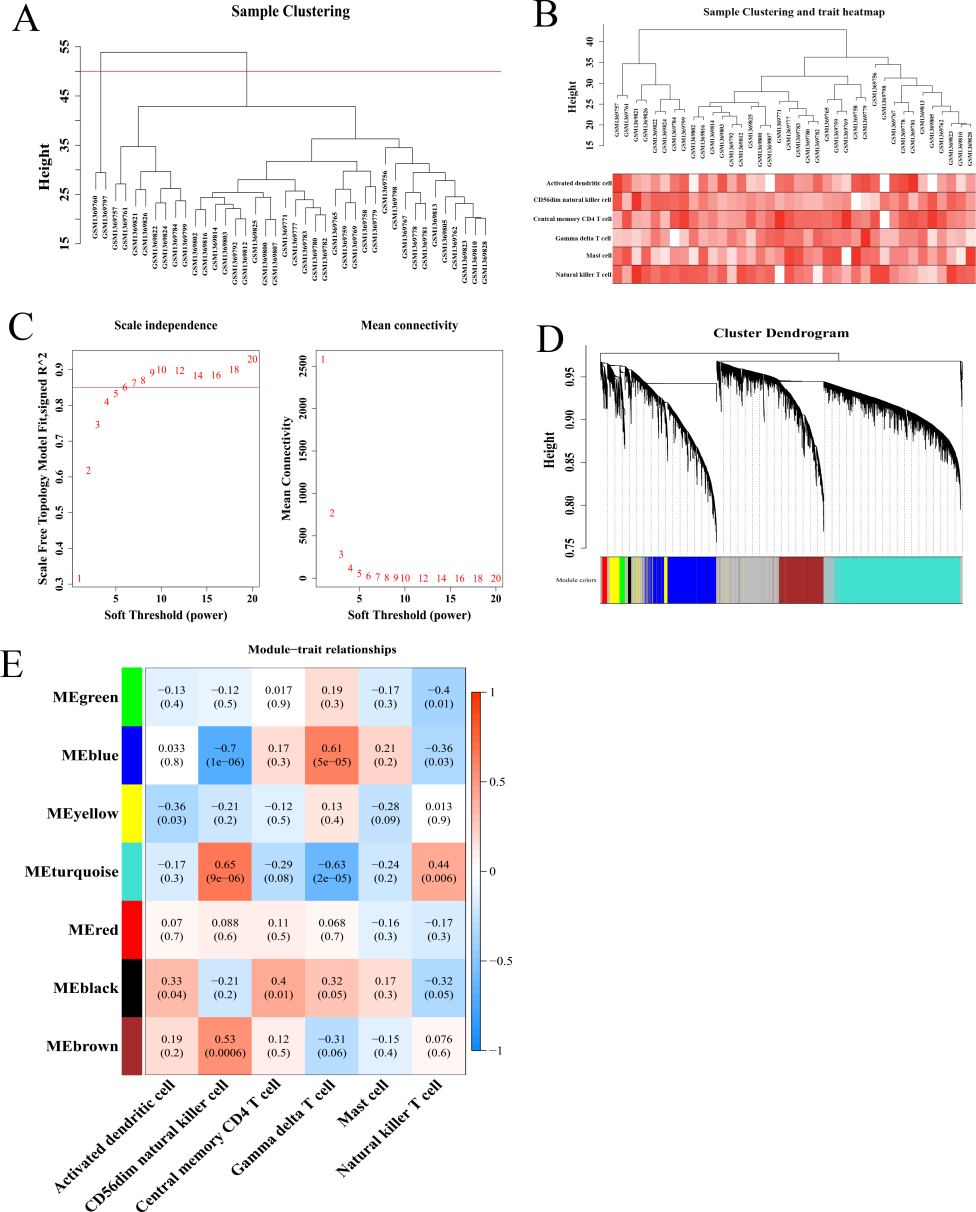
Identification of immune cell-related genes by WGCNA. This analysis was based on the expression profile of human peripheral blood mononuclear cells from the GSE56815 dataset. **(A)** All samples are clustered, and the Euclidean distance of the expression quantity is used for hierarchical clustering (Before removing samples). **(B)** The outlier samples were removed, and the Euclidean distance of expression quantity was re-used for hierarchical clustering, and the trait information of samples was introduced. **(C)** Select the appropriate soft threshold power to determine the threshold of inter-gene correlation. **(D)** WGCNA analysis identified co-expression modules. **(E)** The correlation coefficient matrix between module eigengenes and phenotypic traits was calculated, and a heatmap of the correlations was generated.

Based on the gene expression dataset GSE56815, we adopted WGCNA analysis for the sake of acquiring the immune cell-related genes. Firstly, 2 outliers were removed and clustered again (**Figures 3A, B**). When the soft threshold β was 6, R^2^ was close to 0.85, and mean connectivity tended to 0 (**Figure 3C**). Then, 7 co-expression modules were obtained in Cluster Dendrogram (**Figure 3D**). Subsequently, 7074 immune cell-related genes were contained in blue and turquoise modules depending on Module-trait relationships (**Figure 3E**).

### 3.3 Extraction of candidate genes (autophagy-immune cell-related DEGs)

**Figure 4:**
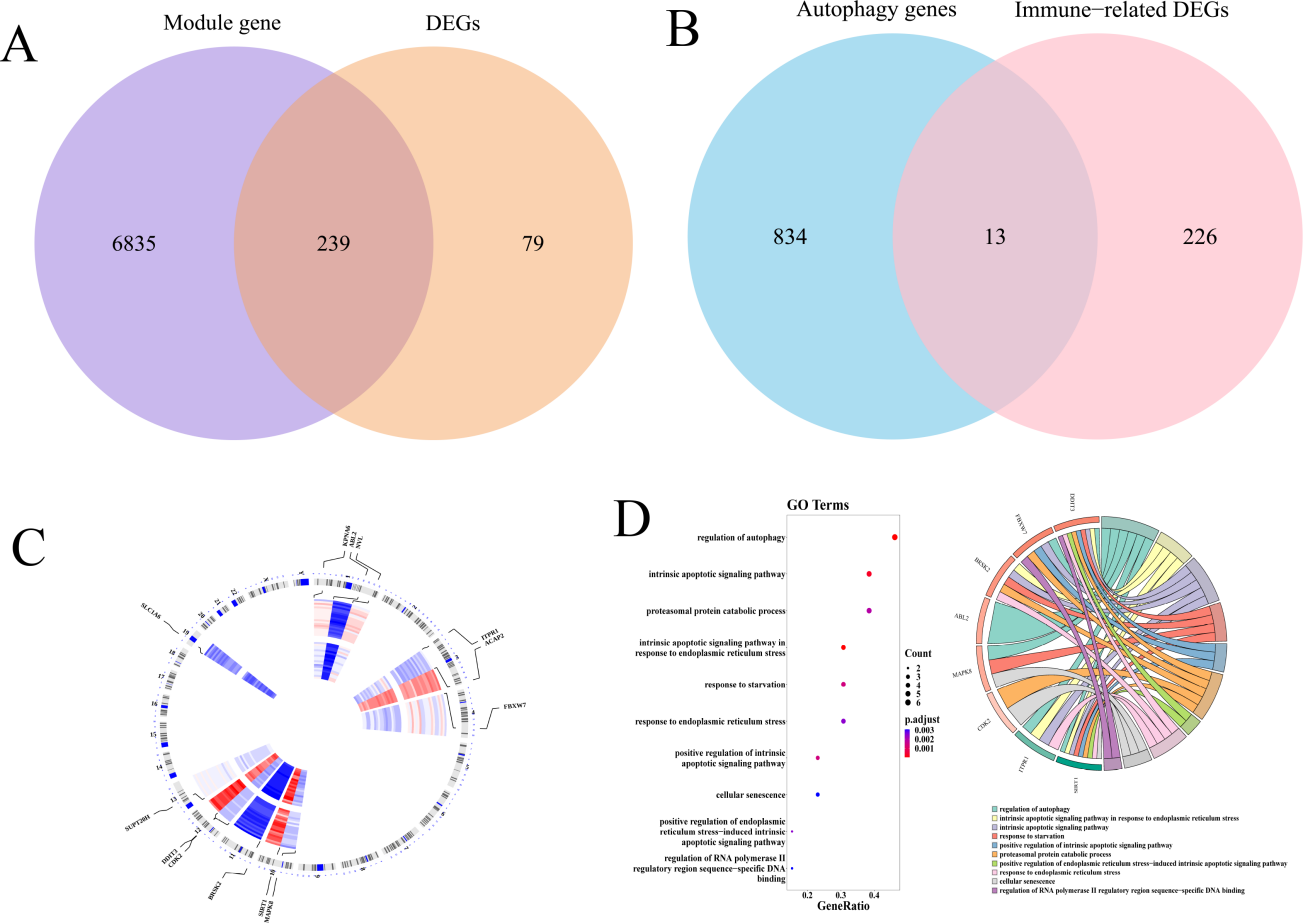
Extraction of candidate genes (autophagy-immune cell-related DEGs). **(A)** Venn diagram showing the intersection between differentially expressed genes and genes in immune-related modules. **(B)** Venn diagram illustrating the intersection between immune-related differentially expressed genes and autophagy-related genes. **(C)** Visualization of the chromosomal locations of candidate genes using the R package OmicCircos. **(D)** Visualization of the top 10 most significant Gene Ontology (GO) biological functions obtained from enrichment analysis.

Firstly, 239 immune cell-related DEGs were acquired by overlapping 318 DEGs and 7074 differential immune cell-related genes (**Figure 4A**). Then, 13 candidate genes (*BRSK2*, *KPNA6*, *ITPR1*, *DDIT3*, *FBXW7*, *NVL*, *CDK2*, *SUPT20H*, *SLC1A6*, *ACAP2*, *SIRT1*, *MAPK8*, and *ABL2*) were extracted by overlapping 239 immune cell-related DEGs and 847 ARGs (**Figure 4B**). **Figure 4C** displayed the chromosome localization of candidate genes.

### 3.4 Functional annotation of candidate genes

**Figure 5:**
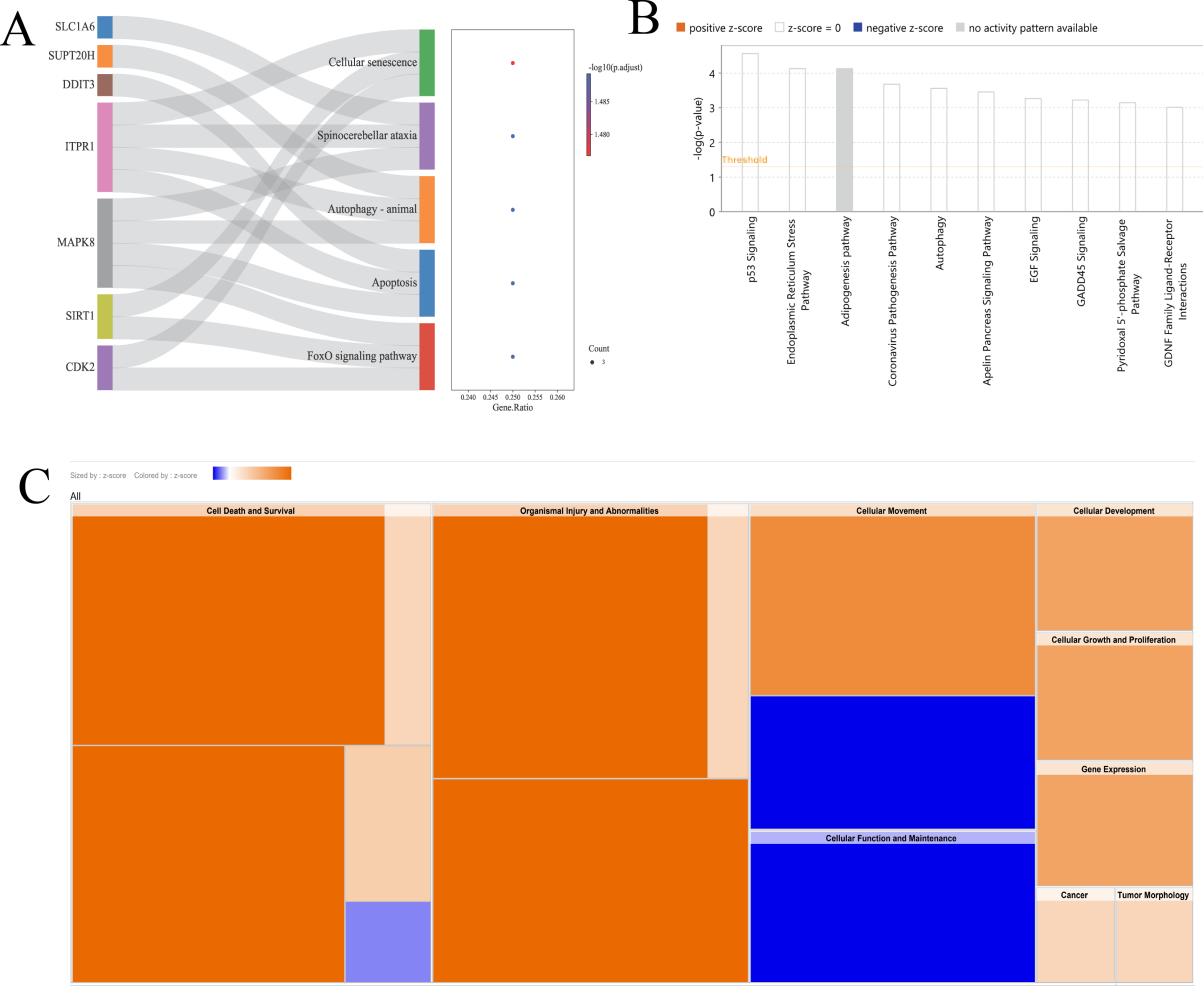
KEGG enrichment analysis and IPA analysis were conducted on immune-autophagy-related differentially expressed genes. **(A)** Visualization of KEGG enrichment results. **(B)** Classical pathway analysis and biological function analysis of candidate genes were conducted using QIAGEN IPA. **(C)** Heatmap visualization of the biological functions of candidate genes analyzed by IPA.

To investigate the function of candidate genes, we performed GO and KEGG analysis. According to GO result, candidate genes were associated with ‘regulation of autophagy’, ‘proteasomal protein catabolic process’, ‘cellular senescence’, ‘response to starvation’ and apoptosis-related pathways (**Figure 4D**). Moreover, in KEGG terms, ‘FoxO signaling pathway’, ‘apoptosis’, ‘autophagy-animal’, and ‘cellular senescence’ were relevant to candidate genes (**Figure 5A**). In addition, autophagy-immune cell-related DEGs were enriched in ‘p53 signaling’, ‘endoplasmic reticulum stress pathway’, ‘ EGF signaling’ and ‘autophagy’ depending on IPA (**Figure 5B**). Finally, candidate genes were positively associated with ‘cell death and survival’ and ‘organismal injury and abnormalities’, and they were negatively correlated with ‘cellular function and maintenance’ (**Figure 5C**).

### 3.5 The establishment of a diagnostic model and nomogram in OP

**Figure 6:**
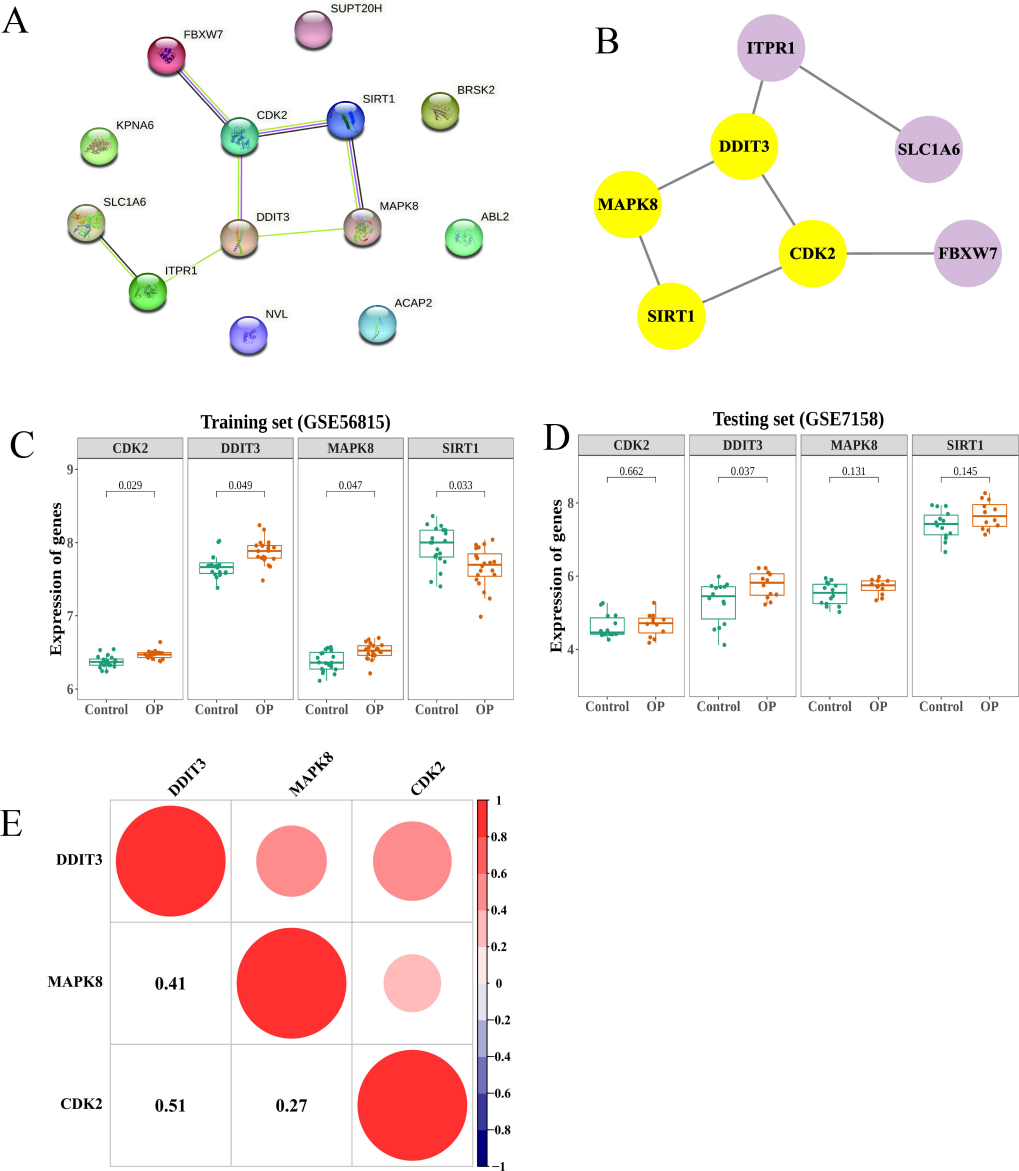
PPI network analysis of candidate genes was performed to further identify key hub genes. **(A)** PPI (protein-protein interaction) network analysis of candidate genes was conducted. **(B)** Core candidate genes, defined as key hub genes, were identified using the MCODE plugin(DDIT3, MAPK8, SIRT1, CDK2). **(C)** Boxplot of expression levels of hub genes in the training set (GSE56815). **(D)** Boxplot of expression levels of hub genes in the validation set (GSE7158). **(E)** Heatmap visualization of the correlations among hub genes.

**Figure 7:**
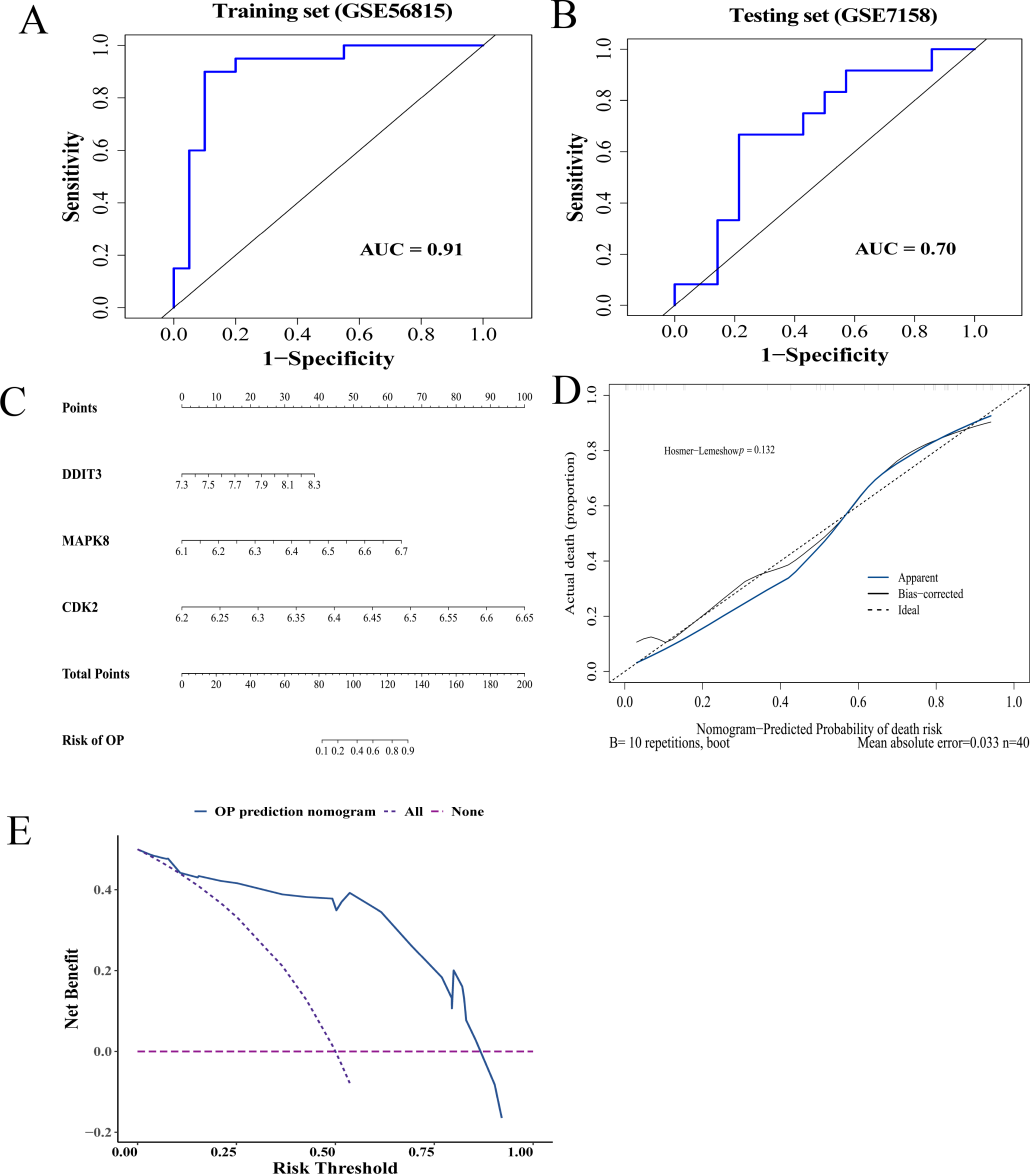
Construction of a logistic regression diagnostic model and evaluation of its performance. **(A)** ROC curve was plotted in the training set (GSE56815) using the R package PRROC. **(B)** ROC curve was plotted in the validation set (GSE7158) using the R package PRROC. **(C)** Constructing a nomogram model based on hub genes. **(D, E)** Calibration curves and decision curves were plotted to reflect the predictive accuracy and clinical applicability of the nomogram model.

To determine the linkage of 13 candidate genes, we constructed a PPI network. Then, 7 candidate genes (*FBXW7*, *CDK2*, *SIRT1*, *MAPK8*, *DDIT3*, *ITPR1*, and *SLC1A6*) linked in the PPI network were certificated (**Figure 6A**). **Figure 6B** indicated 4 critical genes (*DDIT3*, *MAPK8*, *SIRT1*, and *CDK2*) were further selected by MCODE plug-in. Next, we compared the expression discrepancy of critical genes between OP and NC specimens in GSE56815 and GSE7158 datasets. In GSE56815 dataset, *CDK2*, *DDIT3*, and *MAPK8* were highly expressed and *SIRT1* was lowly expressed in OP specimens (**Figure 6C**). Moreover, in GSE7158 dataset, *CDK2*, *DDIT3*, *MAPK8*, and *SIRT1* indicated a trend of high expression in OP specimens (**Figure 6D**). In summary, 3 genes (*CDK2*, *DDIT3*, and *MAPK8*) with consistent expression trends in two datasets were defined as hub genes. Spearman correlation revealed the positive correlation between *DDIT3* and *CDK2*/*MAPK8* was stronger, which indicated *DDIT3* might have a synergistic effect on other hub genes’ expression (**Figure 6E**). Subsequently, we established a diagnostic model for OP depending on the 3 hub genes. The coefficient of hub genes were displayed in **Table 2**. The AUC value of diagnostic model was 0.91, which indicated the assessed value of the model was excellent (**Figure 7A**). Moreover, the AUC value (0.70) of the model in GSE7158 dataset indicated that it was valid (**Figure 7B**). To integrate with the clinical traits of OP patients, a nomogram was established depending on the 3 hub genes (**Figure 7C**). The DCA curve and calibration curve revealed the nomogram had a predictive value (**Figures 7D, E**).

**Table 2:** Logistic regression coefficients of hub genes in the osteoporosis diagnostic model.

Hub gene	DDIT3	MAPK8	CDK2
Coefficients	3.39	9.36	19.49

### 3.6 Functional annotation of hub genes

**Figure 8:**
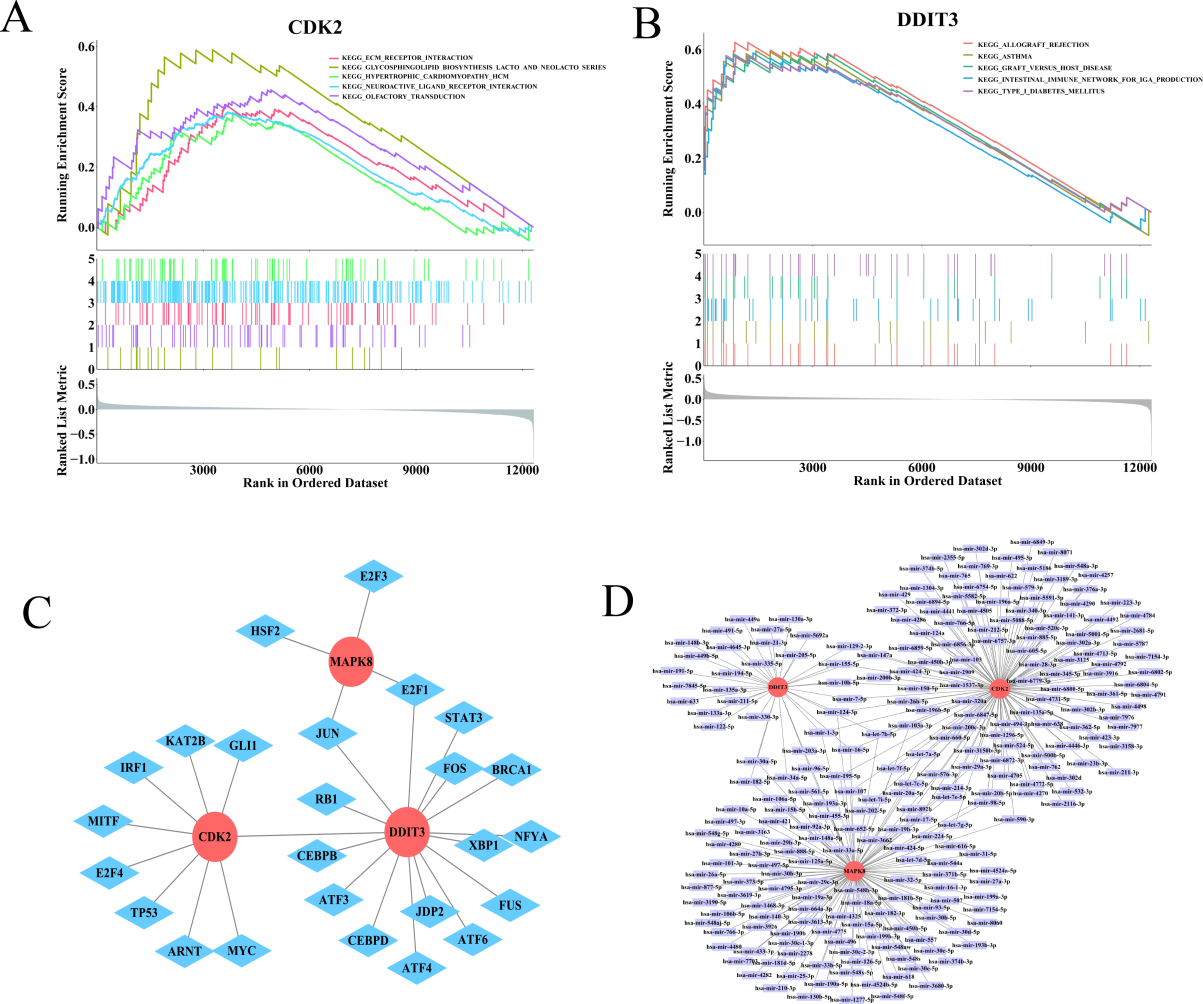
Validation of expression levels of key hub genes using the validation set (GSE7158) and construction of regulatory networks between hub genes and miRNAs, as well as between hub genes and transcription factors (TFs). **(A)** GSEA enrichment analysis of the CDK2 gene. **(B)** GSEA enrichment analysis of the DDIT3 gene. **(C)** Hub gene-transcription factor (TF) regulatory network. **(D)** Hub gene-miRNA regulatory network.

For the purpose of exploring the potential pathways for hub genes, we implemented GSEA analysis. The results demonstrated *CDK2*-high-expression group was associated with ‘ECM receptor interaction’, ‘neuroactive ligand receptor interaction’, and ‘glycophingolipid biosynthesis lacto and neolacto series’ (** 8A**). In addition, *DDIT3*-high-expression group was associated with pathways related to intercellular interactions, ‘antigen processing and presentation’, ‘type I diabetes mellitus’ and ‘hematopoietic cell lineage’ (**Figure 8B**, **Table 3**).

**Table 3:** KEGG Gene Set Enrichment Analysis (GSEA) results of the DDIT3 high-expression group.

ID	Description	setSize	enrichmentScore	NES	pvalue	p.adjust	qvalues	rank	leading_edge	core_enrichment
KEGG_ALLOGRAFT_REJECTION	KEGG_ALLOGRAFT_REJECTION	34	0.626402907836204	1.98642828193197	0.000107498258100348	0.00736427903125424	0.00686231350625935	855	tags=29%, list=7%, signal=27%	3126/3002/3117/3109/3113/3115/959/942/3108/3133
KEGG_ASTHMA	KEGG_ASTHMA	27	0.595796083852226	1.79060196087798	0.00164854675809292	0.0301684056731003	0.0281120605064266	1434	tags=37%, list=12%, signal=33%	3126/3117/3109/3113/3115/8288/959/3108/6356/2206
KEGG_INTESTINAL_IMMUNE_NETWORK_FOR_IGA_PRODUCTION	KEGG_INTESTINAL_IMMUNE_NETWORK_FOR_IGA_PRODUCTION	42	0.587649971920703	1.9542120594052	0.000120725885758266	0.00736427903125424	0.00686231350625935	1354	tags=36%, list=11%, signal=32%	3126/3117/3109/7852/8741/3113/7040/9020/3115/959/23495/942/3108/4055/2826
KEGG_GRAFT_VERSUS_HOST_DISEASE	KEGG_GRAFT_VERSUS_HOST_DISEASE	37	0.582448955003567	1.8742288921988	0.000581439905308167	0.0177339171118991	0.0165251341508637	3417	tags=62%, list=28%, signal=45%	3126/3002/3117/3109/3113/3115/942/3108/3133/3824/3134/3553/3802/57292/3123/3135/356/3122/3569/3106/3811/3119/3803
KEGG_TYPE_I_DIABETES_MELLITUS	KEGG_TYPE_I_DIABETES_MELLITUS	40	0.577022945598737	1.89506522986575	0.00020986851010305	0.00938476985714877	0.00874508321459937	879	tags=25%, list=7%, signal=23%	3126/3002/3117/3109/3113/3115/942/3108/3133/3630
KEGG_SYSTEMIC_LUPUS_ERYTHEMATOSUS	KEGG_SYSTEMIC_LUPUS_ERYTHEMATOSUS	52	0.572365474360975	1.99370678661157	0.0000303934692717474	0.00556200487672978	0.00518288633897167	2383	tags=50%, list=19%, signal=41%	3126/2214/3117/3109/712/2215/3113/2212/89/3115/727/959/720/942/3108/2903/716/2213/713/9103/3586/717/732/3123/718/88
KEGG_AUTOIMMUNE_THYROID_DISEASE	KEGG_AUTOIMMUNE_THYROID_DISEASE	47	0.540217700616084	1.85334910304884	0.000896400889584478	0.0234344803991371	0.0218371344530354	2698	tags=40%, list=22%, signal=32%	3126/3002/3117/3109/3113/3115/959/942/3108/3133/3134/3452/3586/3123/3444/3135/356/3122/3441
KEGG_HEMATOPOIETIC_CELL_LINEAGE	KEGG_HEMATOPOIETIC_CELL_LINEAGE	85	0.46920363360108	1.76513397333282	0.000256414476971278	0.00938476985714877	0.00874508321459937	2218	tags=33%, list=18%, signal=27%	3126/2322/911/290/3563/912/928/7850/2811/1436/945/914/3581/925/929/3678/1438/920/921/7037/1437/930/3553/913/3675/1440/3123/2056
KEGG_ANTIGEN_PROCESSING_AND_PRESENTATION	KEGG_ANTIGEN_PROCESSING_AND_PRESENTATION	79	0.443227202600801	1.65352084446539	0.00140990641230496	0.0301684056731003	0.0281120605064266	3417	tags=46%, list=28%, signal=33%	3126/3117/972/3109/3810/3113/3115/10437/3310/3108/3133/925/3824/3134/5641/3305/3452/920/3802/57292/3123/3444/3135/821/3122/2923/3441/3806/3439/4049/3106/3811/6892/5994/3119/3803
KEGG_CELL_ADHESION_MOLECULES_CAMS	KEGG_CELL_ADHESION_MOLECULES_CAMS	118	0.409764322873997	1.63188671720722	0.00150818861843202	0.0301684056731003	0.0281120605064266	2648	tags=34%, list=21%, signal=27%	3126/3117/3109/3113/3384/914/6404/3115/959/942/6403/4359/3108/3133/53842/925/23705/10666/3134/50848/1002/3383/920/923/5175/3680/5010/1000/3123/1272/6900/3135/54413/9074/23114/4267/9379/80380/7122/3122
KEGG_CYTOKINE_CYTOKINE_RECEPTOR_INTERACTION	KEGG_CYTOKINE_CYTOKINE_RECEPTOR_INTERACTION	236	0.332809855509741	1.45672450938758	0.00252048228980844	0.0419316599122677	0.0390735053539682	1856	tags=23%, list=15%, signal=20%	2322/6352/3563/7850/7132/27242/7852/3561/8741/7292/1436/6358/7040/2833/8795/3627/3587/5196/959/23495/3597/3581/51330/268/5159/658/3579/3625/4283/1232/944/6356/8740/1438/8809/6366/4055/3467/8995/4050/64109/2826/1956/27190/10563/3452/729230/56034/64806/1437/7048/3553/3586/6354

### 3.7 The network of hub genes and TFs/miRNAs

To investigate the regulatory mechanisms of hub genes, we established the network between hub genes and TFs/miRNAs. The hub gene-TF network (28 edges and 28 nodes) contained 3 hub genes and 25 TFs, where JUN and E2F1 could bind specifically to *MAPK8* and *DDIT3* (**Figure 8C**). Moreover, the hub gene-miRNA network (250 nodes and 284 edges) contained 3 hub genes and 247 miRNAs, where hsa-mir-124-3p and hsa-let-7b-5p could regulate the expression of *DDIT3*, *CDK2*, and *MAPK8* (**Figure 8D**).

### 3.8 The immune characteristics and therapeutic drugs of OP

**Figure 9:**
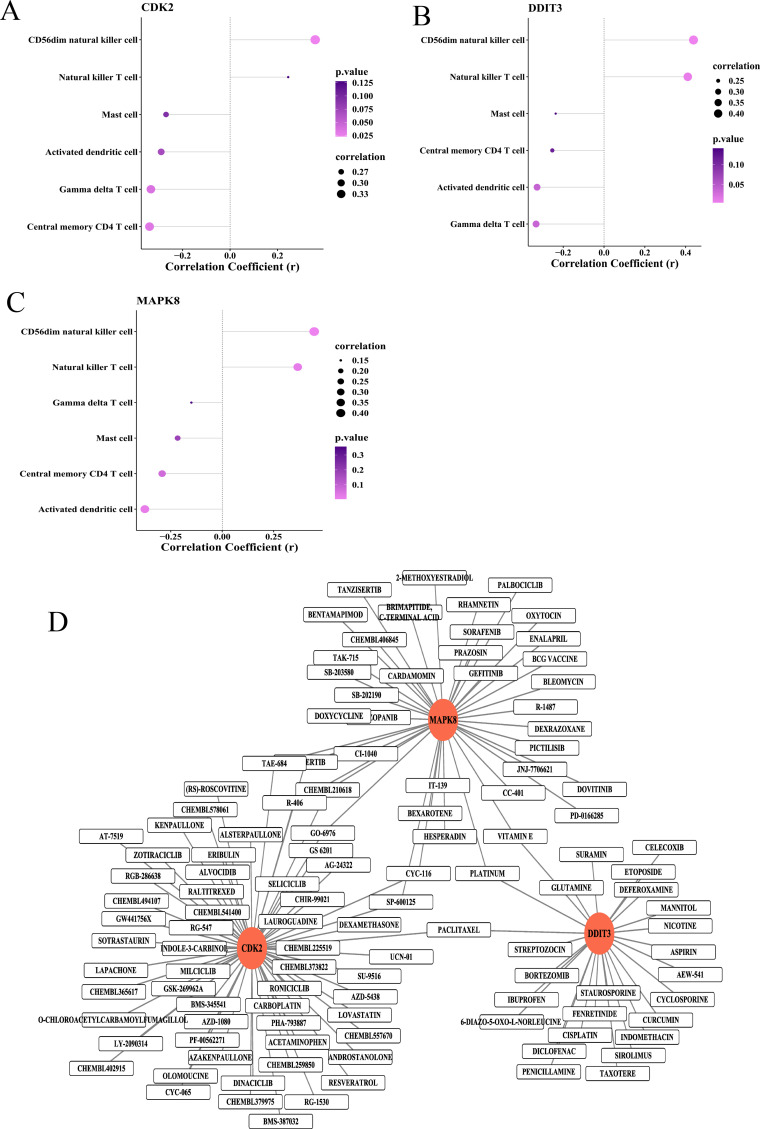
Small molecule drug prediction and correlation analysis between hub gene and immune infiltrating cells were performed. **(A)** Correlation between CDK2 and differential immune infiltrating cells. **(B)** Correlation between DDIT3 and differential immune infiltrating cells. **(C)** Correlation between MAPK8 and differential immune infiltrating cells. **(D)** Prediction of small molecule drugs corresponding to hub genes using the DGIdb database (https://dgidb.org/).

To figure out the relevance between OP and immune cells, we researched the correlation of 3 hub genes (*CDK2*, *DDIT3*, and *MAPK8*) with 6 differential immune cells acquired from GSVA package. The results displayed 3 hub genes were positively associated with CD56dim natural killer cell, and *DDIT3* and *MAPK8* were positively correlated with natural killer T cell (**Figures 9A–C**). Moreover, *DDIT3* and *MAPK8* were negatively correlated with activated dendritic cell, and *CDK2* and *DDIT3* were negatively correlated with gamma delta T cell (**Figures 9A–C**). To develop new therapeutic targets for OP, we performed drug prediction. In total, 61 drugs targeted *CDK2*, 26 drugs targeted *DDIT3*, and 40 drugs targeted *MAPK8* were extracted (**Figure 9D**). Drug RG-547 and RGB-286638 were inhibitor of *CDK2*, and BENTAMAPIMOD and CC-401 were inhibitor of *MAPK8* (**Table 4**).

**Table 4:** List of candidate small molecule drugs targeting hub genes.

search_term	match_term	match_type	gene	drug	interaction_types	sources	pmids
CDK2	CDK2	Definite	CDK2	PHA-793887	inhibitor	ChemblInteractions|TTD	
CDK2	CDK2	Definite	CDK2	DEXAMETHASONE		NCI	10867026
CDK2	CDK2	Definite	CDK2	CHEMBL402915		DTC	18063365
CDK2	CDK2	Definite	CDK2	AZD-5438	inhibitor	ChemblInteractions|TTD	
CDK2	CDK2	Definite	CDK2	O-CHLOROACETYLCARBAMOYLFUMAGILLOL		NCI	8012959
CDK2	CDK2	Definite	CDK2	ALSTERPAULLONE		DTC	
CDK2	CDK2	Definite	CDK2	CHEMBL379975		DTC	
CDK2	CDK2	Definite	CDK2	DINACICLIB	inhibitor	DTC|MyCancerGenome|ClearityFoundationClinicalTrial|ChemblInteractions|CancerCommons|MyCancerGenomeClinicalTrial	23600925
CDK2	CDK2	Definite	CDK2	CYC-065	inhibitor	TTD	
CDK2	CDK2	Definite	CDK2	SU-9516	inhibitor	ChemblInteractions	10592235|11752352|17139284|17016423
CDK2	CDK2	Definite	CDK2	OLOMOUCINE	inhibitor	DTC	10592235|11752352|17139284|17016423
CDK2	CDK2	Definite	CDK2	BMS-387032	inhibitor	TdgClinicalTrial|ChemblInteractions|CancerCommons|TTD	
CDK2	CDK2	Definite	CDK2	AT-7519	inhibitor	DTC|TdgClinicalTrial|ChemblInteractions|TTD	10592235|18656911
CDK2	CDK2	Definite	CDK2	CHIR-99021		DTC	
CDK2	CDK2	Definite	CDK2	CHEMBL210618		DTC	
CDK2	CDK2	Definite	CDK2	RG-1530		DTC	
CDK2	CDK2	Definite	CDK2	KENPAULLONE		DTC	
CDK2	CDK2	Definite	CDK2	RGB-286638	inhibitor	ChemblInteractions|TTD	
CDK2	CDK2	Definite	CDK2	SELICICLIB	inhibitor	DTC|MyCancerGenome|TdgClinicalTrial|ChemblInteractions|TTD	17108108|21080703
CDK2	CDK2	Definite	CDK2	ALVOCIDIB	inhibitor	MyCancerGenome|TdgClinicalTrial|ChemblInteractions	18465538|11752352
CDK2	CDK2	Definite	CDK2	GS 6201		DTC	
CDK2	CDK2	Definite	CDK2	CHEMBL373822		DTC	17181166
CDK2	CDK2	Definite	CDK2	AZAKENPAULLONE		DTC	
CDK2	CDK2	Definite	CDK2	INDOLE-3-CARBINOL		NCI	15611077
CDK2	CDK2	Definite	CDK2	LAUROGUADINE		DTC	
CDK2	CDK2	Definite	CDK2	GW441756X		DTC	
CDK2	CDK2	Definite	CDK2	SP-600125		DTC	
CDK2	CDK2	Definite	CDK2	CHEMBL557670		DTC	19410453
CDK2	CDK2	Definite	CDK2	PF-00562271		DTC	
CDK2	CDK2	Definite	CDK2	GO-6976		DTC	
CDK2	CDK2	Definite	CDK2	ZOTIRACICLIB	inhibitor	DTC|ChemblInteractions|TTD	22148278
CDK2	CDK2	Definite	CDK2	MILCICLIB	inhibitor	DTC|ChemblInteractions	19603809
CDK2	CDK2	Definite	CDK2	RALTITREXED		NCI	10047461
CDK2	CDK2	Definite	CDK2	CHEMBL259850		DTC	
CDK2	CDK2	Definite	CDK2	LAPACHONE		NCI	12689523
CDK2	CDK2	Definite	CDK2	AG-24322	inhibitor	ChemblInteractions|TTD	
CDK2	CDK2	Definite	CDK2	RG-547	inhibitor	ChemblInteractions	10592235
CDK2	CDK2	Definite	CDK2	CYC-116		DTC	
CDK2	CDK2	Definite	CDK2	CHEMBL541400		DTC	
CDK2	CDK2	Definite	CDK2	RESVERATROL		NCI	15122319
CDK2	CDK2	Definite	CDK2	RONICICLIB	inhibitor	ChemblInteractions	
CDK2	CDK2	Definite	CDK2	CHEMBL494107		DTC	17643111
CDK2	CDK2	Definite	CDK2	ANDROSTANOLONE		NCI	10698512
CDK2	CDK2	Definite	CDK2	ACETAMINOPHEN		NCI	14644624
CDK2	CDK2	Definite	CDK2	LY-2090314		DTC	
CDK2	CDK2	Definite	CDK2	BMS-345541		DTC	
CDK2	CDK2	Definite	CDK2	SOTRASTAURIN		DTC	
CDK2	CDK2	Definite	CDK2	(RS)-ROSCOVITINE		DTC	
CDK2	CDK2	Definite	CDK2	CHEMBL365617		DTC	
CDK2	CDK2	Definite	CDK2	CENISERTIB		DTC	
CDK2	CDK2	Definite	CDK2	UCN-01	inhibitor	ChemblInteractions	
CDK2	CDK2	Definite	CDK2	CHEMBL225519		DTC	
CDK2	CDK2	Definite	CDK2	LOVASTATIN		NCI	9553123
CDK2	CDK2	Definite	CDK2	CHEMBL578061		DTC	
CDK2	CDK2	Definite	CDK2	PACLITAXEL		NCI	16020661
CDK2	CDK2	Definite	CDK2	R-406		DTC	
CDK2	CDK2	Definite	CDK2	TAE-684		DTC	
CDK2	CDK2	Definite	CDK2	GSK-269962A		DTC	
CDK2	CDK2	Definite	CDK2	AZD-1080		DTC	
CDK2	CDK2	Definite	CDK2	ERIBULIN		CIViC	26006067
CDK2	CDK2	Definite	CDK2	CARBOPLATIN		CIViC	26006067
DDIT3	DDIT3	Definite	DDIT3	FENRETINIDE		NCI	12234979|17273769
DDIT3	DDIT3	Definite	DDIT3	PACLITAXEL		NCI	8554977
DDIT3	DDIT3	Definite	DDIT3	DICLOFENAC		NCI	15131590
DDIT3	DDIT3	Definite	DDIT3	SURAMIN		NCI	9586958
DDIT3	DDIT3	Definite	DDIT3	TAXOTERE		NCI	10470115
DDIT3	DDIT3	Definite	DDIT3	CISPLATIN		NCI	10071988|8554977
DDIT3	DDIT3	Definite	DDIT3	STAUROSPORINE		NCI	17167033
DDIT3	DDIT3	Definite	DDIT3	CYCLOSPORINE		NCI	15494209
DDIT3	DDIT3	Definite	DDIT3	VITAMIN E		NCI	15271854
DDIT3	DDIT3	Definite	DDIT3	CELECOXIB		NCI|PharmGKB	17167033|17166886|15131590|16597647|22336956
DDIT3	DDIT3	Definite	DDIT3	SIROLIMUS		NCI	11145585
DDIT3	DDIT3	Definite	DDIT3	STREPTOZOCIN		NCI	8482431
DDIT3	DDIT3	Definite	DDIT3	NICOTINE		NCI	11592233
DDIT3	DDIT3	Definite	DDIT3	INDOMETHACIN		NCI	15131590
DDIT3	DDIT3	Definite	DDIT3	MANNITOL		NCI	8670069
DDIT3	DDIT3	Definite	DDIT3	PLATINUM		NCI	8996528
DDIT3	DDIT3	Definite	DDIT3	ASPIRIN		NCI	10688535
DDIT3	DDIT3	Definite	DDIT3	DEFEROXAMINE		NCI	8794898
DDIT3	DDIT3	Definite	DDIT3	ETOPOSIDE		NCI	9044846
DDIT3	DDIT3	Definite	DDIT3	GLUTAMINE		NCI	10377266
DDIT3	DDIT3	Definite	DDIT3	CURCUMIN		NCI	15271854
DDIT3	DDIT3	Definite	DDIT3	IBUPROFEN		NCI	15131590
DDIT3	DDIT3	Definite	DDIT3	BORTEZOMIB		NCI	16357160
DDIT3	DDIT3	Definite	DDIT3	PENICILLAMINE		NCI	11526215
DDIT3	DDIT3	Definite	DDIT3	6-DIAZO-5-OXO-L-NORLEUCINE		NCI	10377266
DDIT3	DDIT3	Definite	DDIT3	AEW-541		CIViC	28637688
MAPK8	MAPK8	Definite	MAPK8	JNJ-7706621		DTC	
MAPK8	MAPK8	Definite	MAPK8	OXYTOCIN		NCI	11566737
MAPK8	MAPK8	Definite	MAPK8	TANZISERTIB	inhibitor	ChemblInteractions|TTD	
MAPK8	MAPK8	Definite	MAPK8	CYC-116		DTC	
MAPK8	MAPK8	Definite	MAPK8	CENISERTIB		DTC	
MAPK8	MAPK8	Definite	MAPK8	SORAFENIB		PharmGKB	20124951
MAPK8	MAPK8	Definite	MAPK8	BRIMAPITIDE, C-TERMINAL ACID		TdgClinicalTrial|TTD	
MAPK8	MAPK8	Definite	MAPK8	GO-6976		DTC	
MAPK8	MAPK8	Definite	MAPK8	CHEMBL406845		DTC	
MAPK8	MAPK8	Definite	MAPK8	DOXYCYCLINE		NCI	16202406|15728539
MAPK8	MAPK8	Definite	MAPK8	CC-401	inhibitor	ChemblInteractions|TTD	
MAPK8	MAPK8	Definite	MAPK8	SB-202190		DTC	22951114
MAPK8	MAPK8	Definite	MAPK8	BCG VACCINE		NCI	14742634
MAPK8	MAPK8	Definite	MAPK8	PALBOCICLIB		DTC	
MAPK8	MAPK8	Definite	MAPK8	PLATINUM		NCI	12181446
MAPK8	MAPK8	Definite	MAPK8	DEXRAZOXANE		NCI	10487526
MAPK8	MAPK8	Definite	MAPK8	PAZOPANIB		DTC	
MAPK8	MAPK8	Definite	MAPK8	BENTAMAPIMOD	inhibitor	ChemblInteractions	
MAPK8	MAPK8	Definite	MAPK8	GEFITINIB		NCI	17205515
MAPK8	MAPK8	Definite	MAPK8	R-406		DTC	
MAPK8	MAPK8	Definite	MAPK8	CARDAMOMIN		DTC	25959811
MAPK8	MAPK8	Definite	MAPK8	PRAZOSIN		NCI	12095132
MAPK8	MAPK8	Definite	MAPK8	VITAMIN E		NCI	16909121
MAPK8	MAPK8	Definite	MAPK8	BEXAROTENE		NCI	17027148
MAPK8	MAPK8	Definite	MAPK8	PICTILISIB		DTC	
MAPK8	MAPK8	Definite	MAPK8	R-1487		DTC	
MAPK8	MAPK8	Definite	MAPK8	TAE-684		DTC	
MAPK8	MAPK8	Definite	MAPK8	BLEOMYCIN		NCI	16148050
MAPK8	MAPK8	Definite	MAPK8	SP-600125		DTC	
MAPK8	MAPK8	Definite	MAPK8	CHEMBL210618		DTC	
MAPK8	MAPK8	Definite	MAPK8	RHAMNETIN		DTC	24397781
MAPK8	MAPK8	Definite	MAPK8	TAK-715		DTC	
MAPK8	MAPK8	Definite	MAPK8	DOVITINIB		DTC	
MAPK8	MAPK8	Definite	MAPK8	PD-0166285		DTC	
MAPK8	MAPK8	Definite	MAPK8	ENALAPRIL		NCI	16772709
MAPK8	MAPK8	Definite	MAPK8	IT-139		TTD	
MAPK8	MAPK8	Definite	MAPK8	SB-203580		DTC	
MAPK8	MAPK8	Definite	MAPK8	2-METHOXYESTRADIOL		NCI	9647742
MAPK8	MAPK8	Definite	MAPK8	CI-1040		TTD	
MAPK8	MAPK8	Definite	MAPK8	HESPERADIN	inhibitor	DTC	19035792

### 3.9 Identification of key cells

**Figure 10:**
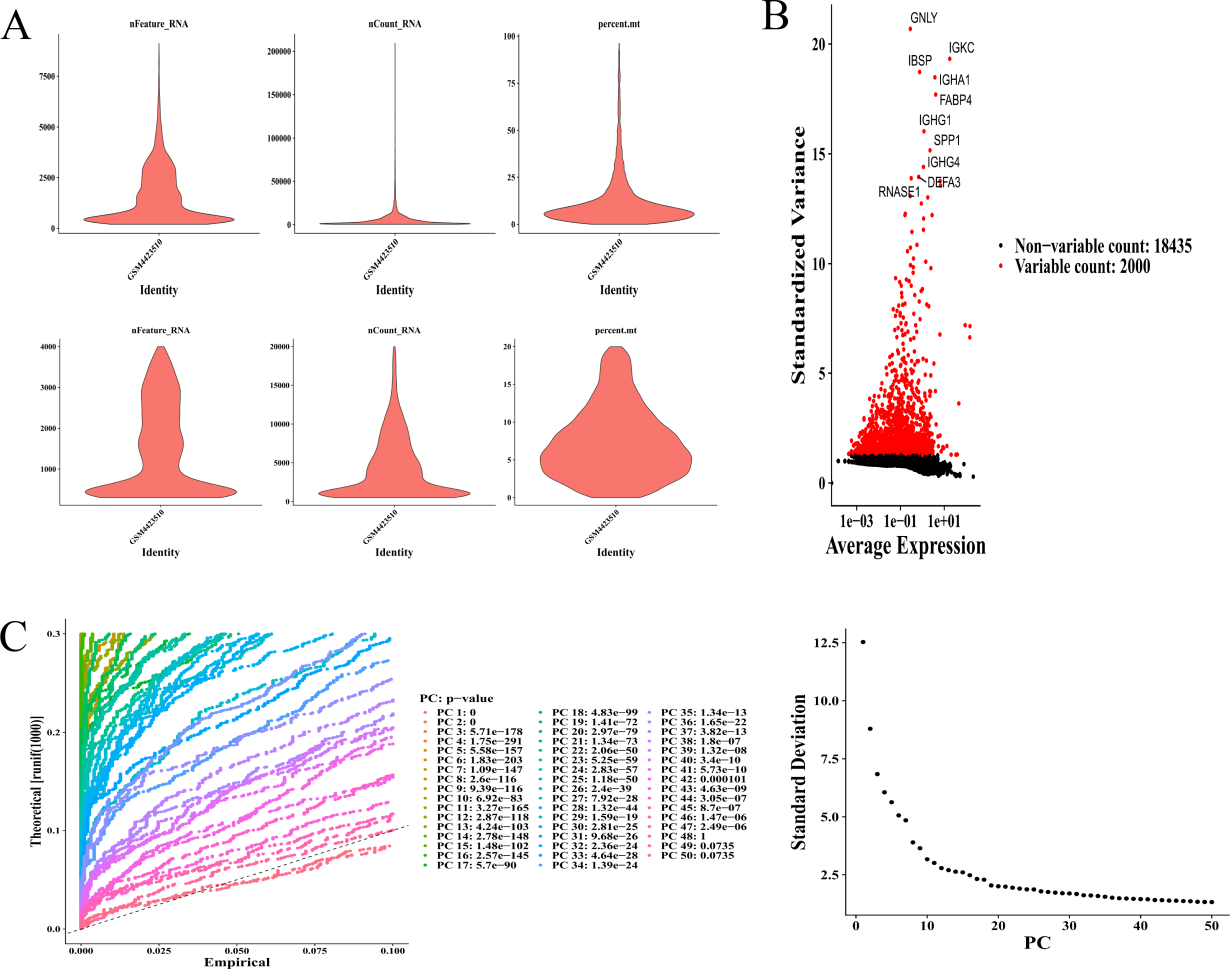
Single-cell RNA sequencing (scRNA-seq) analysis in the GSE147287 dataset. **(A)** Violin plots showing the distribution of quality control metrics (nCount_RNA, nFeature_RNA, percent.mt) in scRNA-seq data before and after filtering. **(B)** Identification of highly variable genes (HVGs), with red indicating HVGs and black indicating non-variable genes. **(C)** Selection of principal components (PCs).

**Figure 11:**
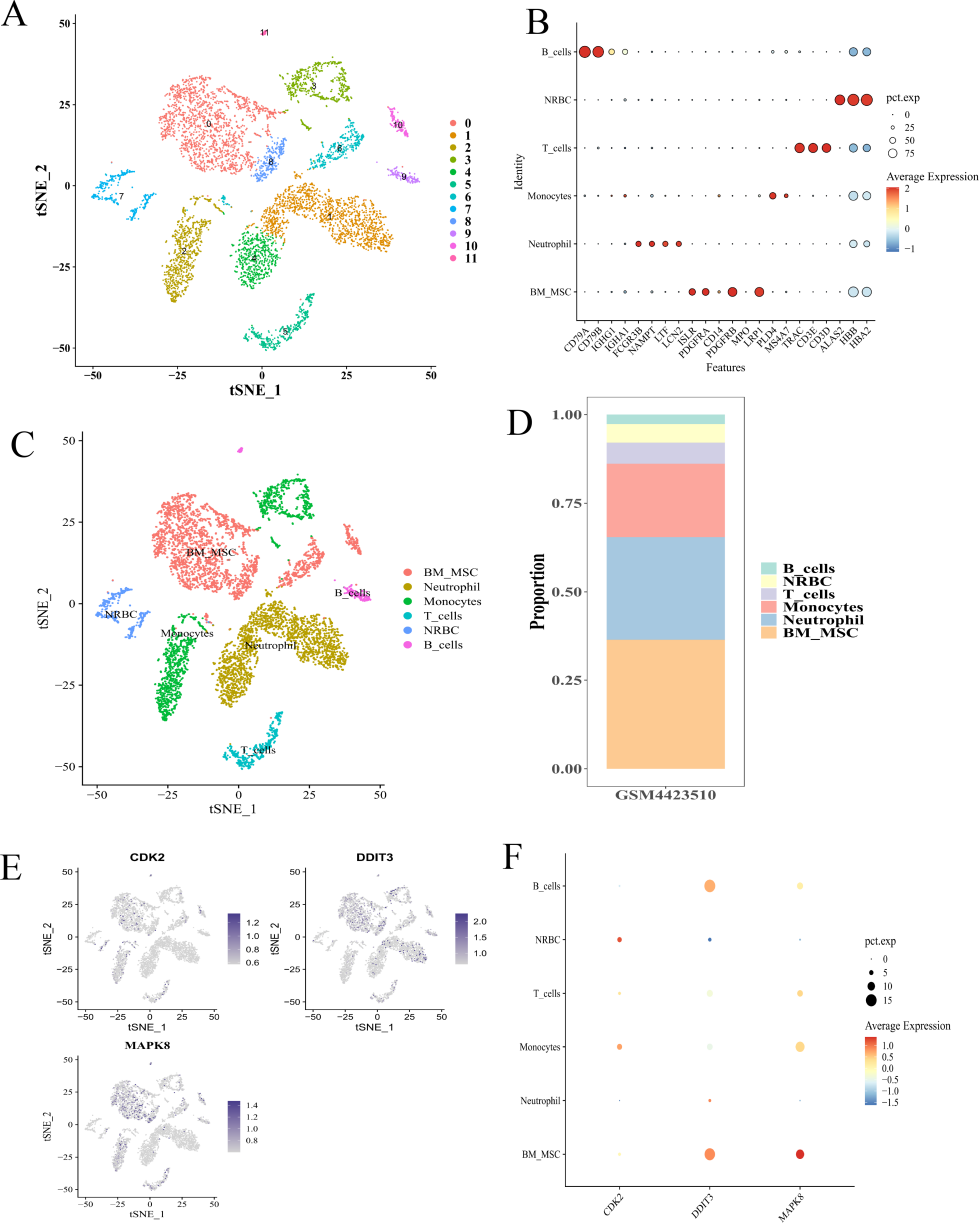
Identification of key cells by scRNA-seq analysis. **(A)** T-distributed Stochastic Neighbor Embedding (tSNE) plot showing 12 clusters (0-11). **(B)** Bubble chart of expression of marker genes in different annotated cells. **(C)** tSNE plot showing 6 annotated cells. **(D)** Proportion of annotated cells in OP samples. **(E)** tSNE plot of hub gene expression in annotated cells. **(F)** Bubble diagram of hub gene expression in annotated cells.

After filtering out ineligible cells and genes, a total of 6,878 cells and 20,435 genes remained (**Figure 10A**). Subsequently, 2,000 HVGs were identified (**Figure 10B**). PCA was performed, selecting the top 15 PCs for downstream analysis (P < 0.05) (**Figure 10C**). Using UMAP, the cells were clustered into 12 distinct groups (resolution = 0.2) (**Figure 11A**). These clusters were annotated as various cell types, including neutrophils, nucleated red blood cells (NRBC), monocytes, T cells, B cells, and bone marrow mesenchymal stem cells (BM MSCs) (**Figures 11B, C**). Among these cell types, BM MSCs represented the largest proportion in the OP sample (**Figure 11D**). In BM MSCs, the genes DDIT3 and MAPK8 exhibited high expression (**Figures 11E, F**). Consequently, BM MSCs were identified as key players in this context.

### 3.10 Exploration of interactions and differentiation trajectories of key cells

**Figure 12:**
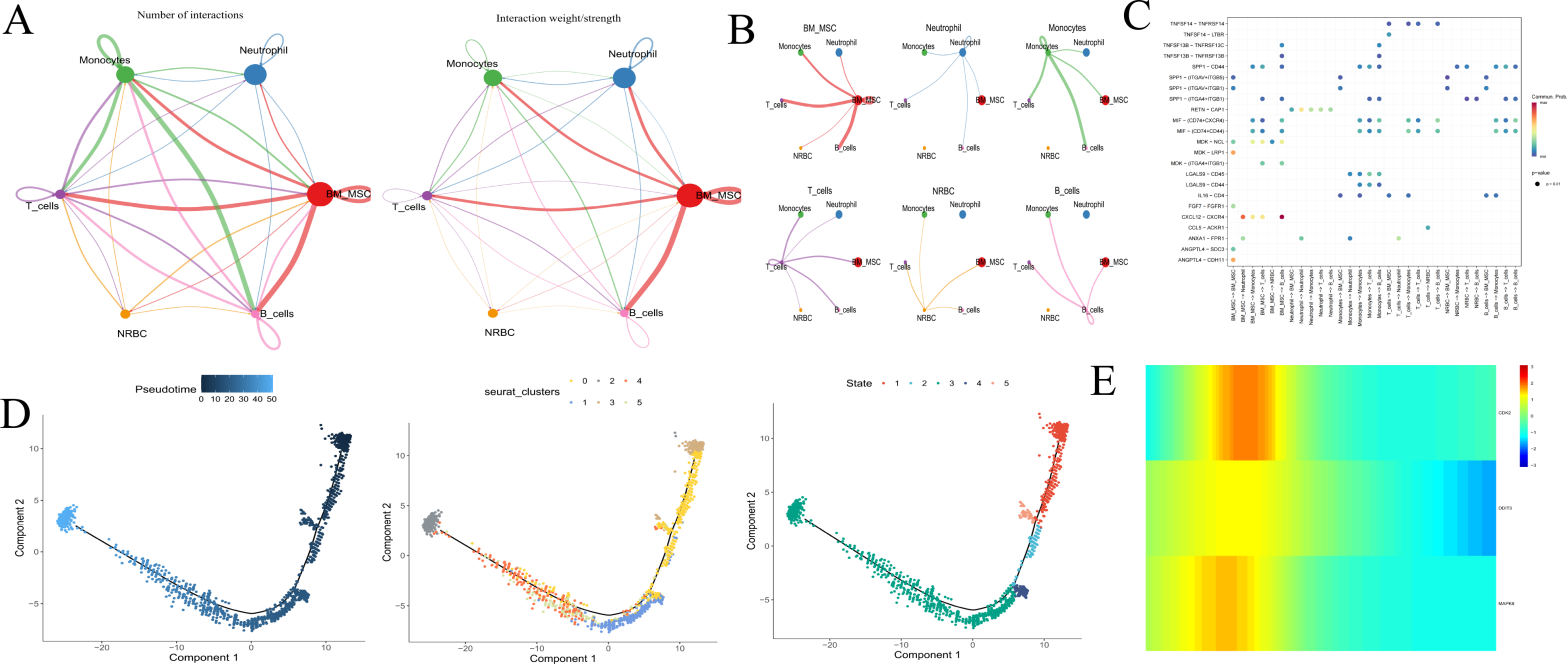
Cell-to-cell communication and pseudo-time analysis of key cells in osteoporosis. **(A)** Cell–cell interaction networks showing the number and strength of interactions in the OP sample. **(B)** Communication networks of each annotated cell type. **(C)** Interaction bubble plots depicting ligand–receptor interactions between key cells and other annotated cell types. **(D)** Differentiation trajectory of bone marrow mesenchymal stem cells (BM MSCs) divided into five stages (Stage 1-5) by pseudo-time analysis. **(E)** Dynamic expression patterns of hub genes (CDK2, DDIT3, MAPK8) across the pseudo-time progression of BM MSCs.

Cell-to-cell communication analysis showed frequent interactions between key cells and other annotated cell types in OP sample (**Figures 12A, B**). Among these, BM MSCs had the more frequent communication with B cells. Additionally, the highest communication probability between BM MSCs and B cells was observed, with the ligand-receptor pair being CXCL12 - CXCR4. (**Figure 12C**).

**Figure 13:**
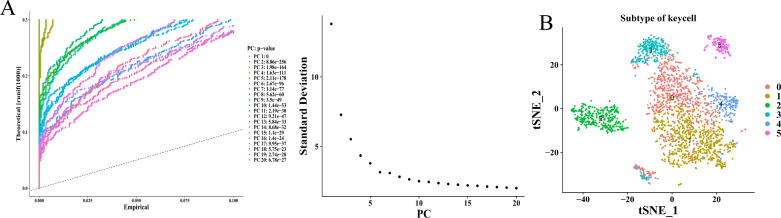
Secondary dimensionality reduction analysis of key cells. **(A)** PCA analysis results. **(B)** tSNE plot of key cells subclusters.

Besides, BM MSCs were divided into 6 subclusters (**Figures 13A, B**). Pseudo-time analysis revealed the differentiation trajectory of BM MSCs, progressing from right (dark blue) to left (light blue), with cells clearly divided into 5 stages (Stage 1-5) (**Figure 12D**). Stage 1 represented the earliest stage of differentiation. During this process, the expressions of the CDK2, DDIT3, MAPK8 were higher in the early and middle stages (**Figure 12E**), suggesting that these genes might not be directly involved in the differentiation process of BM MSCs.

### 3.11 Alterations in bone structure and serum markers in the OVX-OP model group

**Figure 14:**
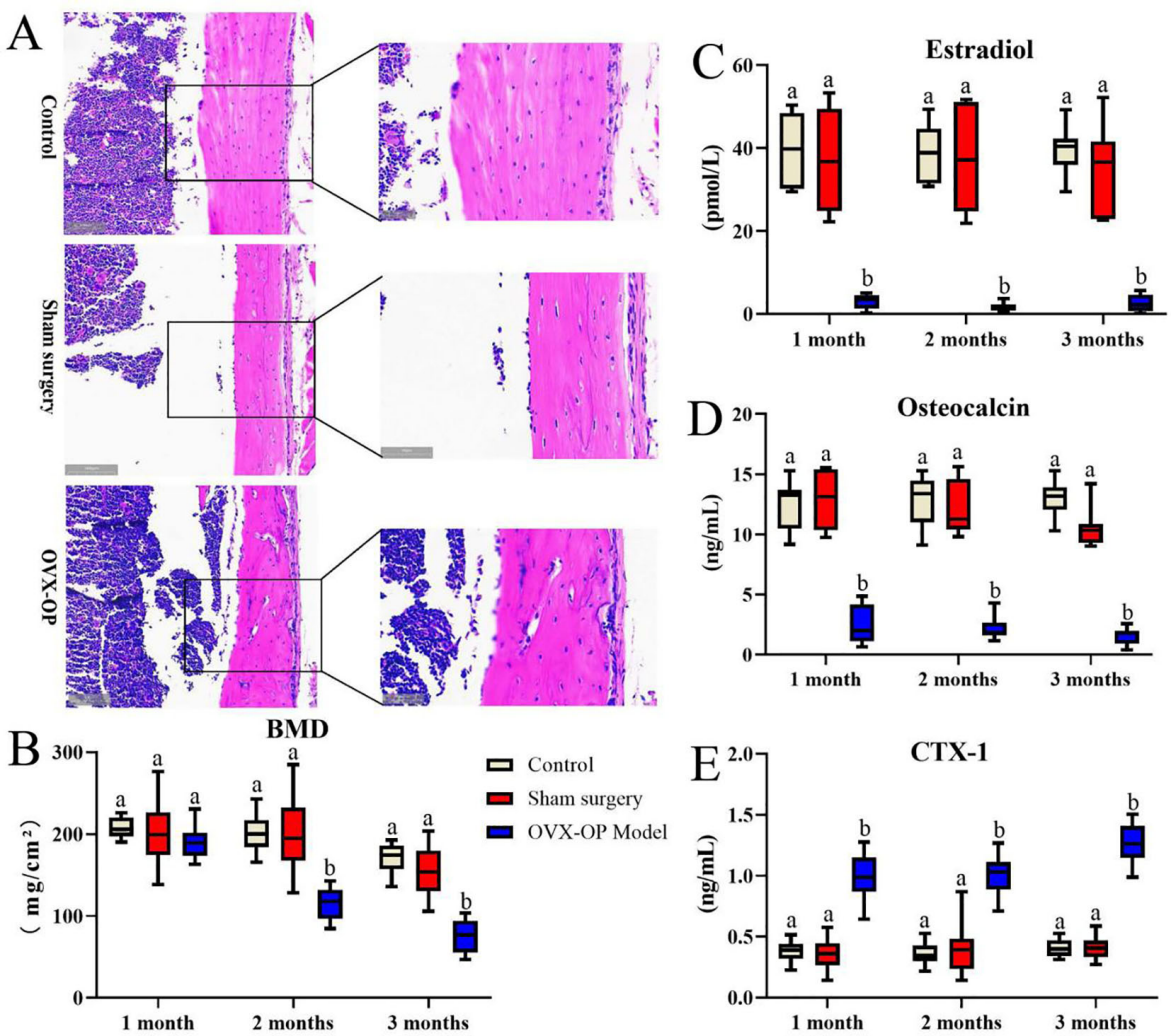
Changes of bone structure and serum markers in OVX-OP model group to verify the model replication results. **(A)** HE staining was used to detect bone tissue sections of mice and observe the changes of bone morphology. **(B)** Bone mineral density (BMD) of the femur was measured using digital X-ray radiometry (Discovery WA, Hologic, USA). **(C–E)** Serum markers estradiol **(C)**, osteocalcin **(D)** and CTX-1 **(E)** associated with bone metabolism were detected by ELISA kit.

HE staining revealed that the OVX-OP model group exhibited a disordered arrangement in bone tissue, indicating a notable fibrosis phenomenon. There was a noticeable decrease in the number of osteoblasts and an increase in the number of osteoclasts along the bone surfaces. The trabecular bone in the OVX group exhibited significant thinning and fragmentation (**Figure 14A**). Additionally, the OVX-OP model group demonstrated a significantly lower bone mineral density (mg/cm^2^) compared to the sham surgery group at 2–3 months post-OVX (P < 0.05) (**Figure 14B**). Furthermore, this model group also showed significantly reduced serum estradiol concentrations (pmol/L) (**Figure 14C**) and lower serum osteocalcin concentrations (ng/mL) (**Figure 14D**) in comparison to the sham surgery group (P < 0.05). Conversely, the OVX-OP model group had significantly higher serum CTX-1 concentrations (ng/mL) at the 1–3 months post-OVX (P < 0.05) (**Figure 14E**). Overall, these findings indicated significant alterations in bone metabolism and serum markers levels in the OVX-OP model group.

### 3.12 Expression verification

**Figure 15:**
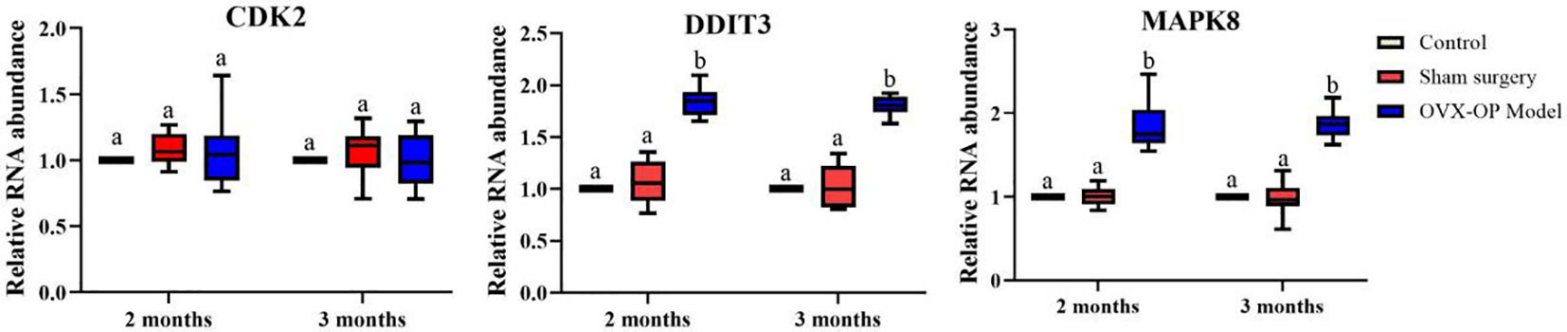
Realtime-PCR analysis for gene expression of CDK2, DDIT3 and MAPK8 in peripheral blood cells of mice.

**Figure 16:**
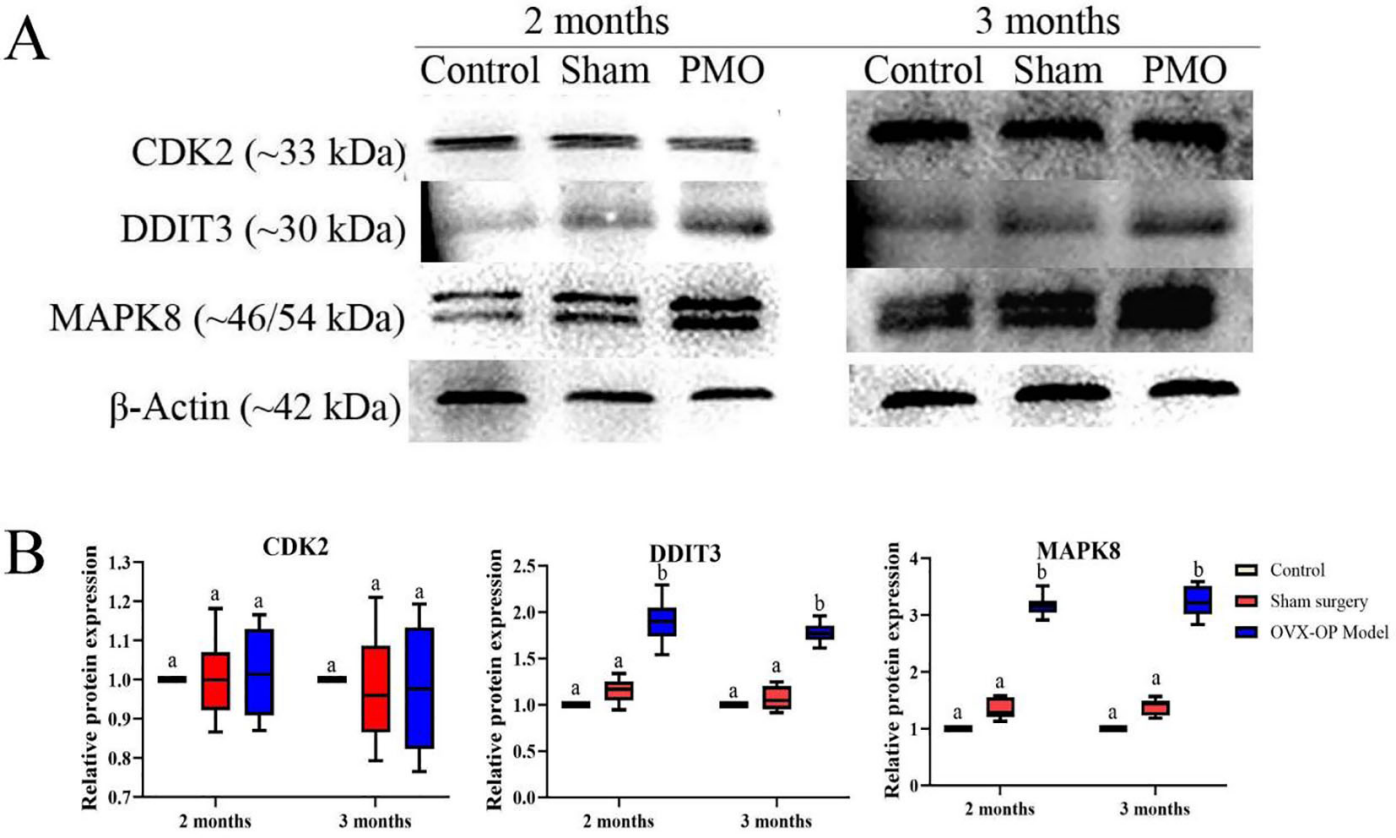
Protein expression of CDK2, DDIT3, and MAPK8 in control, Sham, and postmenopausal osteoporosis (PMO) groups. **(A)** Representative Western blot bands of CDK2 (~33 kDa), DDIT3 (~30 kDa), MAPK8 (~46/54 kDa), and the housekeeping protein β-Actin (~42 kDa). **(B)** Densitometric quantification of protein levels normalized to β-Actin. Data are presented as mean ± SEM. *P < 0.05 vs Control group; #P < 0.05 vs Sham group.

Real-time PCR and Western blot analyses were performed to verify the expression levels of hub genes at mRNA and protein levels. Real-time PCR results showed that the mRNA expressions of MAPK8 and DDIT3 were significantly higher in the OVX-OP group than those in the sham surgery group (P<0.05). There was no significant difference in CDK2 mRNA expression between the two groups (**Figure 15**). Consistent with the mRNA expression trends, Western blot analysis demonstrated that the protein expressions of MAPK8 and DDIT3 were markedly up-regulated in the OVX-OP group (P<0.05), while the protein expression of CDK2 showed no significant difference between the sham group and OVX-OP model group (**Figure 16**). These results indicated that the expression patterns of target genes at both mRNA and protein levels were highly consistent, further supporting the reliability of our findings and providing new insights into the treatment and evaluation of PMO.

## 4 Discussion

PMO is becoming a major global health concern, especially with the rising number of postmenopausal women (14). Presently, the primary therapeutic modalities employed in clinical practice exhibit significant limitations, with evidence suggesting an elevated risk of gastrointestinal and cardiovascular adverse events. This poses a considerable challenge to optimizing treatment efficacy and maintaining patients’ quality of life. Recent research has revealed several mechanisms contributing to PMO, highlighting the complex relationship between hormonal changes and cellular activities in bone metabolism (40). Cytokines secreted by immune cells can either enhance or inhibit the differentiation and activity of osteoclasts and osteoblasts, ultimately affecting bone density (41). However, the mechanisms by which immunity and autophagy contribute to PMO require further investigation. Increased autophagy in osteoclasts promotes bone resorption, and optimal levels of autophagy are crucial for balancing bone remodeling. Dysfunction of autophagy has been associated with the development of PMO (42–44). Studies have identified autophagy as a process involved in quality control, metabolism, and innate immunity. Nonetheless, the molecular mechanisms involving the relevant genes of both processes in PMO have not yet been clearly elucidated.

In our study, we aim to connect two important pathways-immune regulation and autophagy-by identifying biomarkers related to PMO. Studying genes related to immune and autophagic processes will improve our understanding of PMO’s pathophysiology. Changes in the immune system have been shown to play a crucial role in the pathophysiology of PMO (45). In our analysis, we identified six different types of immune infiltrating cells-Activated dendritic cells, CD56dim natural killer cells, Central memory CD4 T cells, Gamma delta T cells, Mast cells, and Natural killer T cells-as significant players in the immune landscape of PMO. For example, activated dendritic cells stimulate osteoclastogenesis and regulate bone-resorbing activity by releasing cytokines (46). Similarly, CD56dim natural killer cells are involved in targeting and eliminating dysfunctional osteoblasts, thereby impacting bone remodeling (47). Additionally, the presence of various T cell subsets, such as Central memory CD4 T cells and Gamma delta T cells, has been linked to the modulation of inflammatory responses that could further exacerbate bone loss in PMO (48). This evidence indicates that the dysregulation of these immune cells may significantly contribute to the disease’s development and progression. In this study, we identified Cyclin-dependent kinase 2 (CDK2), DNA Damage-Inducible Transcript 3 (DDIT3), and Mitogen-Activated Protein Kinase 8 (MAPK8) as hub genes related to immune responses and autophagy in PMO through differential expression analysis and validation of expression levels. Furthermore, we performed Gene Set Enrichment Analysis (GSEA) and drug repurposing predictions. Our findings provide new perspectives on the development of innovative therapeutic strategies and potential treatments for PMO.

CDK2 is mainly recognized for its essential role in regulating the cell cycle, especially during the transition from the G1 phase to the S phase (49, 50). However, new evidence indicates that CDK2 also plays a role in adjusting various immune responses (51). It regulates the growth and development of immune cells, which are crucial for maintaining bone homeostasis (51). In the case of PMO, abnormal CDK2 activity may cause an imbalance between osteoblasts and osteoclasts, leading to increased bone resorption rather than formation. This imbalance can exacerbate the decline in bone density observed in postmenopausal women. It is noteworthy that in the OVX animal model, the expression of CDK2 did not show a significant difference, which is not entirely consistent with our observations in human datasets. We speculate that this inconsistency may stem from several aspects. First, species differences may lead to distinct gene expression patterns-human peripheral blood mononuclear cells (PBMCs) and mouse PBMCs exhibit species-specific responses in the regulatory network of the cell cycle (52). Second, although the OVX mouse model mimics estrogen deficiency, it is difficult to fully replicate the chronic immune-metabolic dysregulation process that unfolds over decades in human PMO. Nevertheless, the diagnostic model constructed based on the three hub genes demonstrated good predictive performance in the human dataset (AUC = 0.91) and was validated in an independent dataset (AUC = 0.70), suggesting that CDK2 still holds potential value in the diagnosis of human PMO. Future studies are warranted to further validate its reliability as a biomarker in larger human cohorts and to explore the molecular mechanisms underlying its functional differences between mice and humans.

DDIT3, also known as CHOP, is a key player in the integrated stress response, particularly during endoplasmic reticulum (ER) stress (53, 54). This study found that DDIT3 is upregulated in PMO patients, and Gene Set Enrichment Analysis (GSEA) revealed its close association with immune-related pathways such as “Antigen processing and presentation” and “Hematopoietic cell lineage”, suggesting that DDIT3 may be involved in the osteoimmune regulation of PMO. Yang et al., using a DDIT3/CHOP knockout mouse model and ATDC5 chondrocyte line, confirmed that DDIT3 can directly bind to the SIRT1 promoter and promote its expression, thereby enhancing autophagy activity by inhibiting the AKT signaling pathway, as evidenced by an increased LC3B-II/I ratio and a higher number of autophagosomes and autolysosomes (55). Shirakawa et al. (2004) found that overexpression of DDIT3 in ST-2 mesenchymal cells accelerated and enhanced the formation of mineralized nodules and upregulated the expression of osteocalcin and alkaline phosphatase, suggesting that DDIT3 may promote osteoblast differentiation (56). These findings indicate that the role of DDIT3 in bone metabolism may depend on the cell type, differentiation stage, and the level of endoplasmic reticulum stress.

MAPK8 or JNK (c-Jun N-terminal kinase), is crucial for various cellular processes, including inflammation, apoptosis, and autophagy (57). MAPK8 is activated in response to stress signals and is involved in the signaling cascades associated with immune responses (58). This study found that MAPK8 expression is elevated in PMO patients and is associated with immune cell infiltration. Regarding osteoclast differentiation, research indicates that the JNK signaling pathway is involved in the RANKL-induced osteoclast differentiation process, and JNK inhibitors can significantly suppress osteoclast formation (59, 60). In terms of autophagy regulation, JNK is recognized as a key regulatory molecule for the autophagy process in various cell types (61). Furthermore, JNK plays a crucial role in inflammatory responses by participating in the regulation of pro-inflammatory cytokine expression (62). Additionally, the MAPK8 inhibitor CC-401 holds potential therapeutic value, as it has demonstrated protective effects in a renal injury model (63). Therefore, MAPK8 may represent a critical node linking inflammatory signaling to osteoclast activation.

GSEA results showed that CDK2 was primarily enriched in the extracellular matrix-receptor interaction and neuroactive ligand-receptor interaction pathways, while DDIT3 was enriched in antigen processing and presentation, type 1 diabetes mellitus, and hematopoietic cell lineage pathways. These pathways are directly involved in the pathological progression of PMOP by regulating bone metabolism. The extracellular matrix-receptor interaction pathway, mediated by integrin family receptors, translates physicochemical signals from the extracellular microenvironment into intracellular biochemical reactions, thereby participating in the regulation of osteoblast fate and function (64, 65). In PMOP, estrogen deficiency impairs ECM-integrin signaling, leading to reduced osteogenic differentiation capacity, enhanced osteoclast attachment and bone resorption, and consequently, trabecular bone destruction and decreased bone mineral density (BMD) (66–68). The high expression of DDIT3 and its enrichment in the antigen processing and presentation pathway aligns closely with the immune infiltration dysregulation observed in PMOP. Postmenopausal chronic low-grade inflammation leads to abnormal activation of dendritic cells and T cells. Enhanced antigen presentation amplifies inflammatory signals, continuously promotes RANKL secretion, stimulates osteoclastogenesis, and thereby exacerbates bone loss (15, 69). Overall, these pathways likely constitute a complex regulatory network that mediates the imbalance between osteogenesis and osteoclastogenesis, contributing to the onset and development of PMOP.

Furthermore, the pathways identified in this study may have extensive interactions with other metabolic and inflammatory diseases. Pathways such as extracellular matrix-receptor interaction, antigen processing and presentation, and those related to diabetes are dysregulated not only in PMOP but also in type 2 diabetes, obesity, rheumatoid arthritis, and chronic kidney disease (70, 71). These diseases share common pathogenic mechanisms, including chronic inflammation, endoplasmic reticulum stress, and autophagy dysfunction, and they may interact through the immune-metabolism-bone axis (71, 72). For example, metabolic disorders in postmenopausal women, synergizing with estrogen deficiency, may aggravate DDIT3-mediated osteoblast apoptosis. Chronic inflammation in autoimmune diseases may enhance immune activation and bone resorption via the antigen presentation pathway. These interactions suggest that CDK2, DDIT3, and MAPK8 could serve as potential common therapeutic targets for the co-morbidities of metabolism, inflammation, and bone loss.

To explore potential therapies targeting these genes, we used the DGIdb database to identify small molecular inhibitors. RG-547 and RGB-286638 are identified as CDK2 inhibitors, while BENTAMAPIMOD and CC-401 are recognized as MAPK8 inhibitors (73). RG-547 and RGB-286638 work by specifically blocking CDK2 activity, which may help reduce abnormal cell cycle progression in osteoblasts and contribute to bone loss (74). In addition, BENTAMAPIMOD and CC-401 target MAPK8, playing a role in modulating inflammatory pathways linked to osteoclast activation (63). Further research on these compounds may reveal new insights into their roles in autophagy modulation, immune regulation, and their potential effectiveness in treating PMO. Therefore, understanding the interplay among immune responses, genetic factors, and potential therapies offers promising opportunities for developing treatment strategies for PMO.

The diagnostic model constructed in this study based on CDK2, DDIT3, and MAPK8 demonstrated good predictive performance in both the training and validation sets (AUC values of 0.91 and 0.70, respectively), suggesting that their combined detection holds potential application value for the auxiliary diagnosis of PMOP. Notably, the expression levels of DDIT3 and MAPK8 were significantly elevated in peripheral blood mononuclear cells from OVX mice, which is consistent with the trend observed in patient data. This indicates that they may serve as dynamic monitoring indicators reflecting disease status. In the future, prospective cohort studies could be designed to evaluate the clinical utility of this gene panel for the early identification of PMOP, prediction of disease progression, and assessment of the efficacy of anti-osteoporotic treatments.

Furthermore, preliminary progress has been made in the research of inhibitors targeting these genes. CDK2 inhibitors, such as RGB-286638, have entered early-stage clinical trials in the field of oncology, showing favorable safety and targeting profiles (74). MAPK8 inhibitors, like CC-401, have also undergone preclinical and exploratory clinical research in inflammation-related diseases such as renal ischemia-reperfusion injury (63), indicating their translational potential. However, the application of these inhibitors in the field of osteoporosis remains unexplored. Therefore, conducting drug repurposing research for bone metabolism using existing inhibitors, or developing novel targeted agents with dual functions in immune regulation and autophagy modulation, will be an important direction for future PMOP therapy.

This study identified three hub genes related to immunity and autophagy-CDK2, DDIT3, and MAPK8-through bioinformatics analysis and explored their potential mechanisms. However, several limitations remain. First, the decision curve analysis of the nomogram indicated limited clinical net benefit, suggesting that the practical application value of this model in real-world clinical settings requires further evaluation. Second, the study was primarily based on transcriptomic data, lacking validation at the protein expression level and functional experiments; thus, the specific regulatory mechanisms of these hub genes in the development and progression of PMO remain unclear. Additionally, the single-cell RNA sequencing dataset used had a small sample size, which may affect the accuracy and generalizability of the results.

To address these limitations, our future research will proceed as follows: First, we will conduct multicenter prospective clinical studies to expand the sample size for validating the clinical utility of the nomogram model, and integrate imaging and biochemical indicators to optimize its predictive performance. Second, we will examine the protein expression levels of the hub genes via Western blot and immunohistochemistry, and employ gene knockout/overexpression cellular models along with conditional knockout mice to further elucidate their molecular regulatory mechanisms. Furthermore, we will integrate larger-scale single-cell sequencing data and spatial transcriptomics to comprehensively reveal the cell-type-specific expression patterns and intercellular communication networks of these hub genes within the bone microenvironment, thereby providing novel targets for the precise diagnosis and treatment of PMO.

## Data Availability

The original contributions presented in the study are included in the article/**Supplementary Material**. Further inquiries can be directed to the corresponding authors.
