# Supplementary figures and images for: Analysis of immune and autophagy-related genes and their regulatory mechanisms in osteoporosis patients post-menopause
Source: Front Endocrinol (Lausanne). 2026 Jun 1;17:1783374. doi: 10.3389/fendo.2026.1783374 (PMC13265317; doi:10.3389/fendo.2026.1783374)

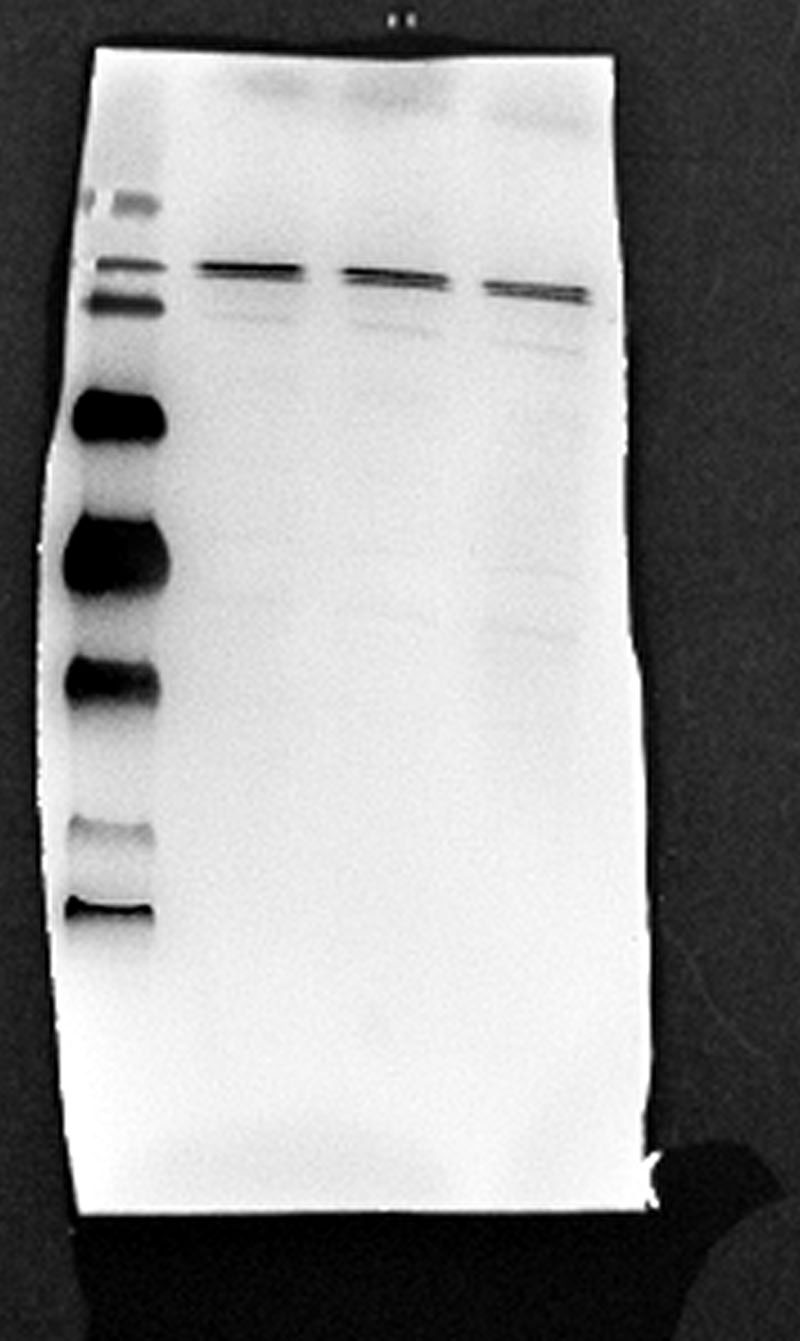

Supplement: Supplementary file 1 [file DataSheet1.zip › WB_Original_2M/CDK2.jpg]

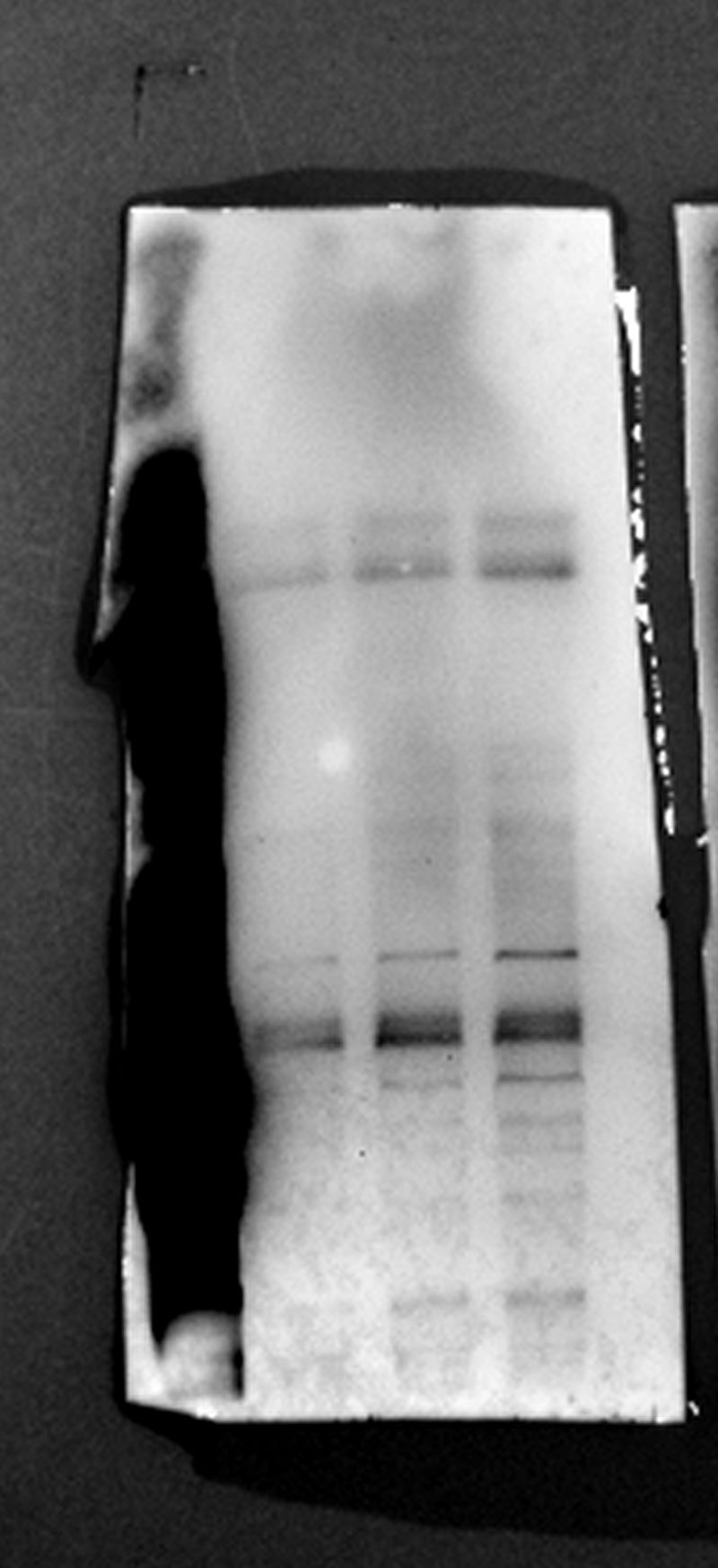

Supplement: Supplementary file 1 [file DataSheet1.zip › WB_Original_2M/DDIT3.jpg]

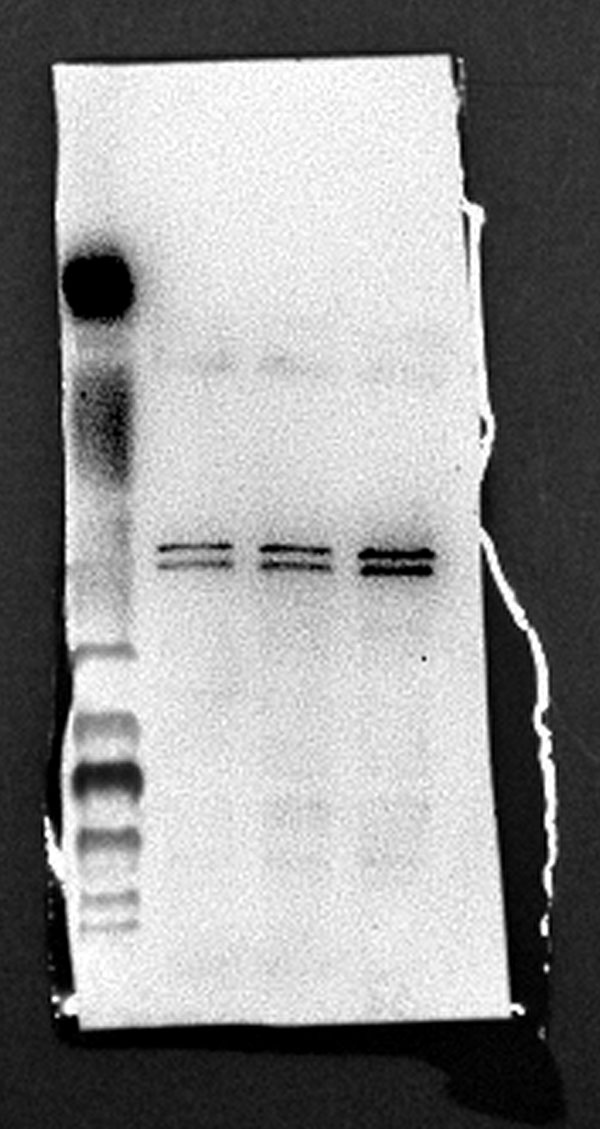

Supplement: Supplementary file 1 [file DataSheet1.zip › WB_Original_2M/MAPK8.jpg]

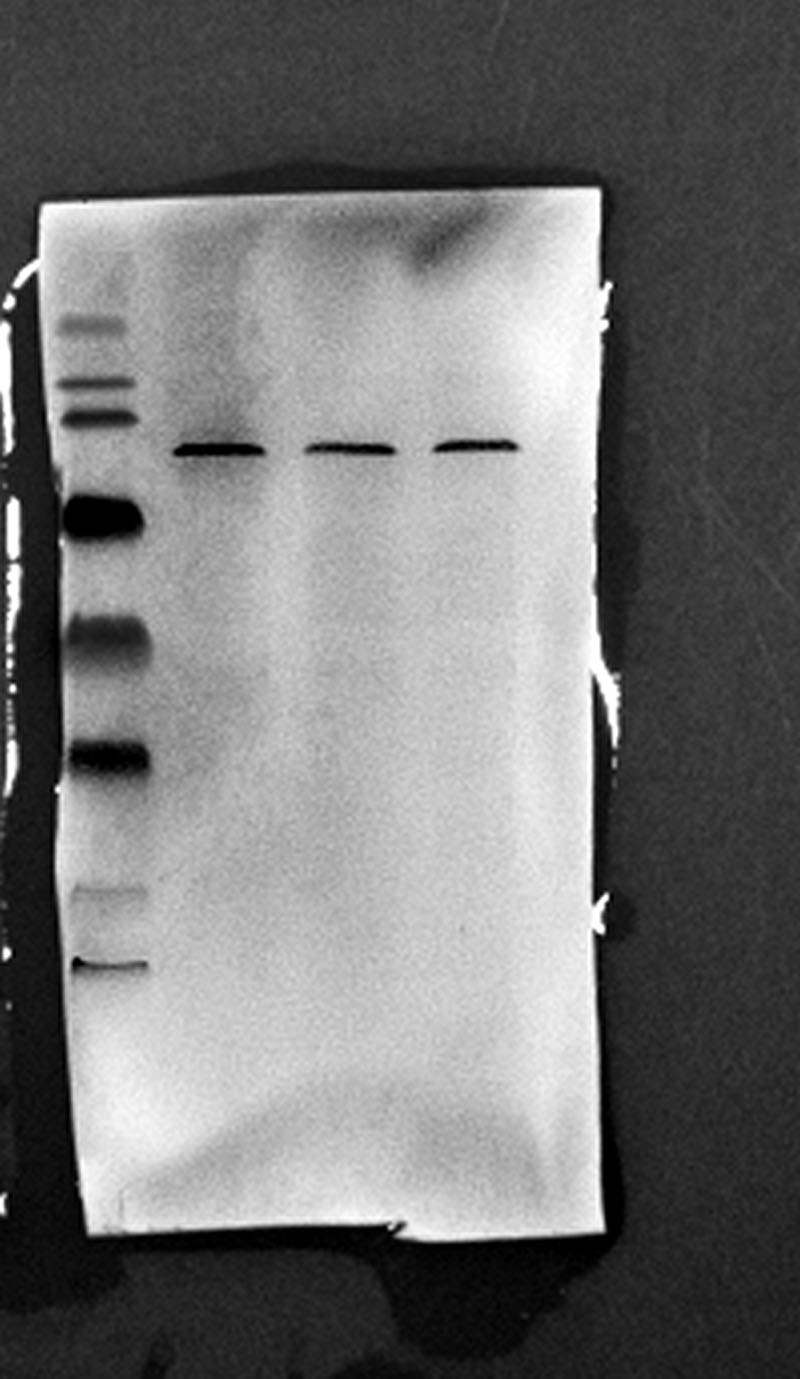

Supplement: Supplementary file 1 [file DataSheet1.zip › WB_Original_2M/β-Actin.jpg]

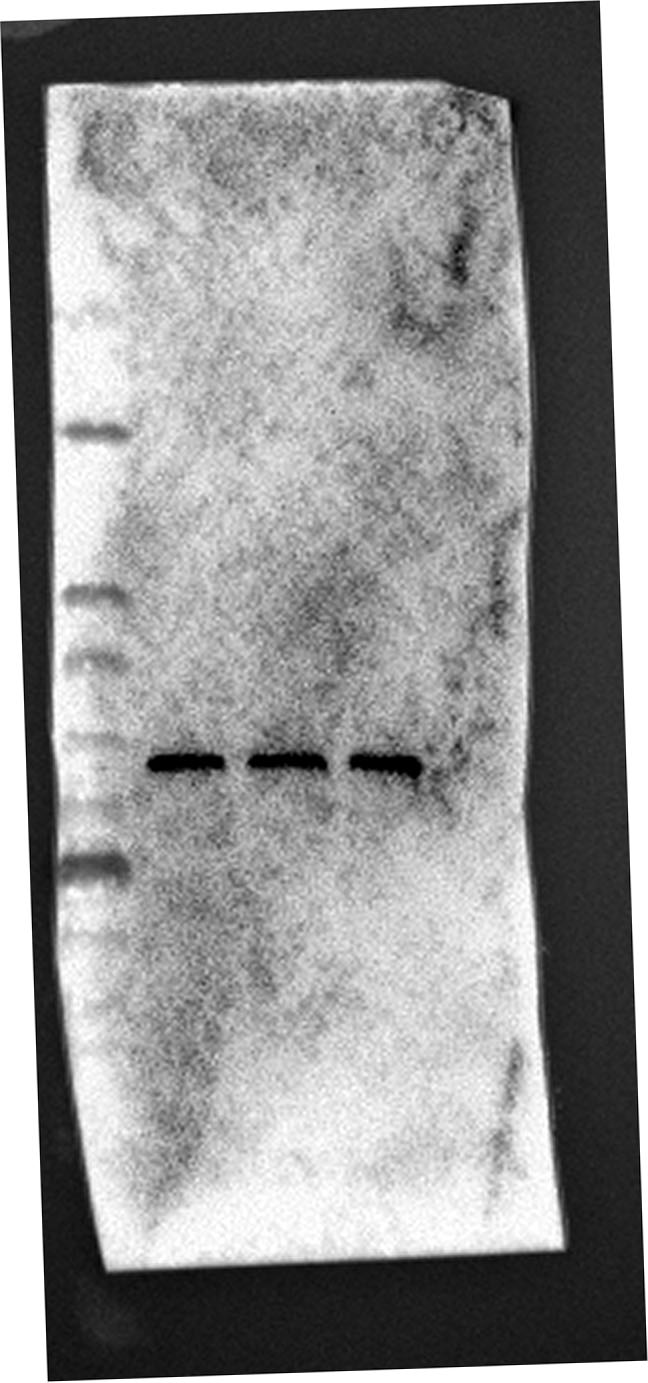

Supplement: Supplementary file 2 [file DataSheet2.zip › WB_Original_3M/CDK2.jpg]

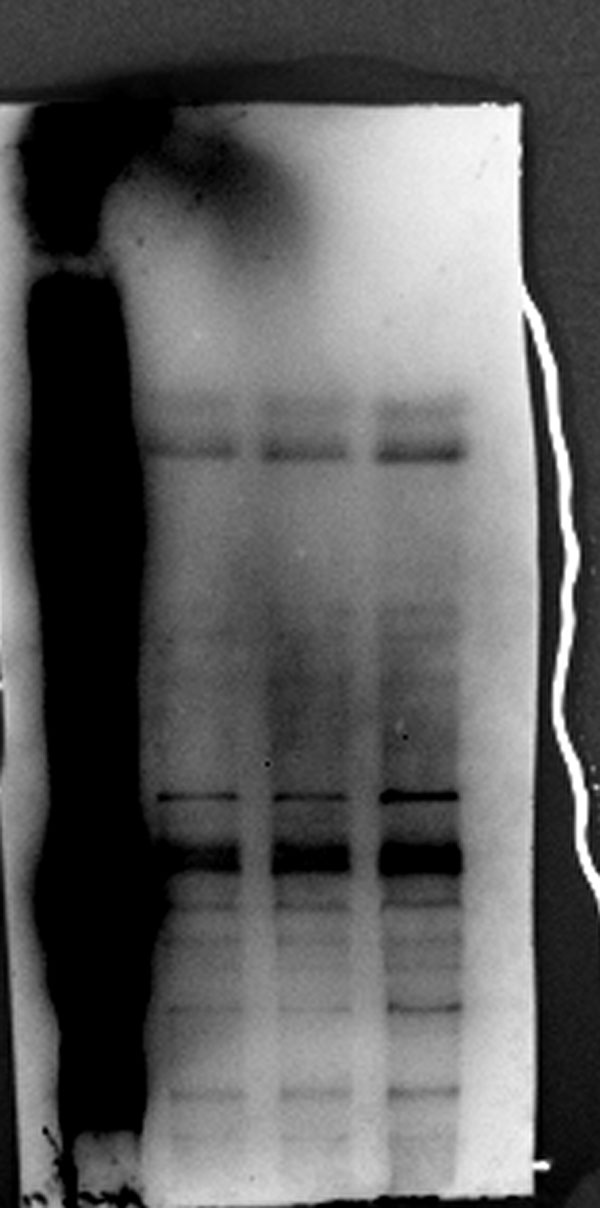

Supplement: Supplementary file 2 [file DataSheet2.zip › WB_Original_3M/DDIT3.jpg]

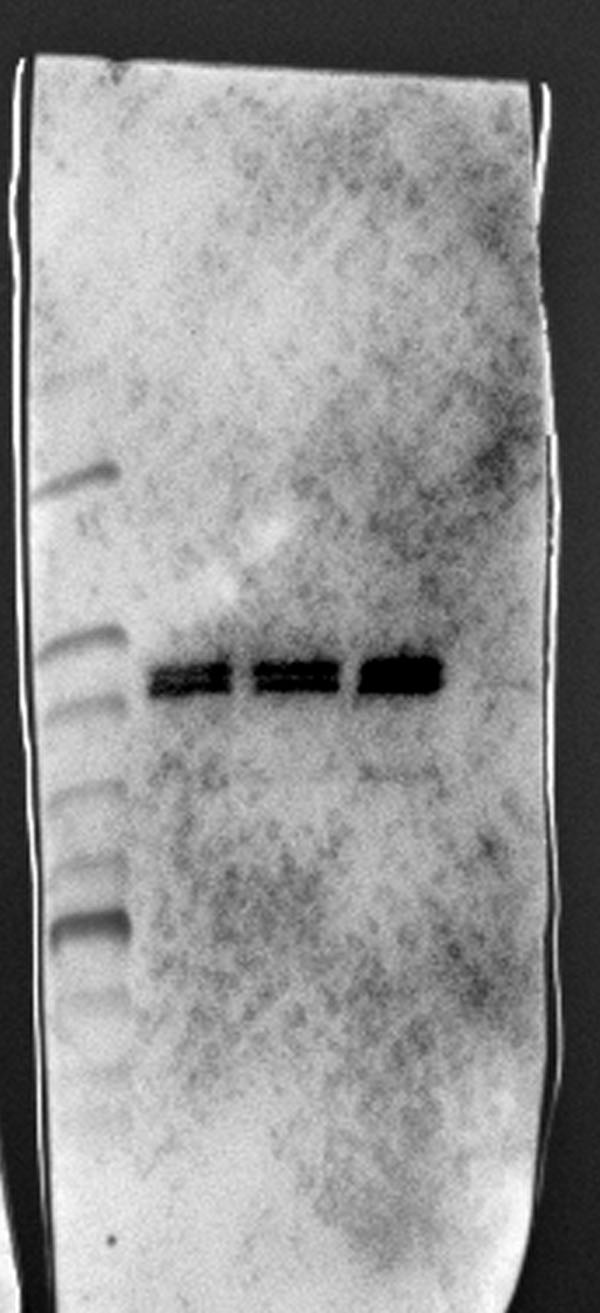

Supplement: Supplementary file 2 [file DataSheet2.zip › WB_Original_3M/MAPK8.jpg]

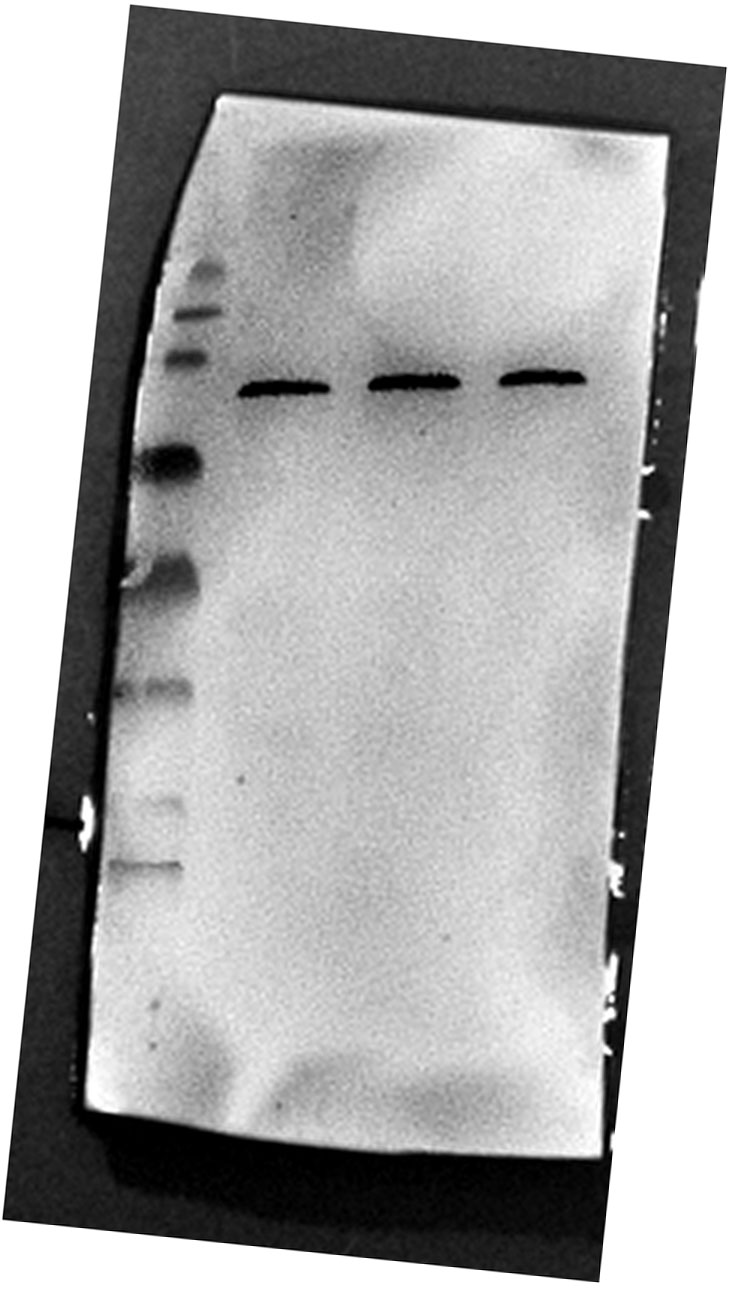

Supplement: Supplementary file 2 [file DataSheet2.zip › WB_Original_3M/β-Actin.jpg]
